# Mapping the Global Distribution of Livestock

**DOI:** 10.1371/journal.pone.0096084

**Published:** 2014-05-29

**Authors:** Timothy P. Robinson, G. R. William Wint, Giulia Conchedda, Thomas P. Van Boeckel, Valentina Ercoli, Elisa Palamara, Giuseppina Cinardi, Laura D'Aietti, Simon I. Hay, Marius Gilbert

**Affiliations:** 1 Livestock Systems and Environment Research Theme (LSE), International Livestock Research Institute (ILRI), Nairobi, Kenya; 2 Animal Production and Health Division (AGA), Food and Agriculture Organization of the United Nations (FAO), Rome, Italy; 3 Environmental Research Group Oxford (ERGO) - Department of Zoology, University of Oxford, Oxford, United Kingdom; 4 Biological Control and Spatial Ecology, Université Libre de Bruxelles, Brussels, Belgium; 5 Fonds National de la Recherche Scientifique, Brussels, Belgium; 6 Department of Ecology and Evolutionary - Biology Department, Princeton University, Princeton, New Jersey, United States of America; 7 Princeton Environmental Institute, Princeton, New Jersey, United States of America; 8 Spatial Ecology and Epidemiology Group - Department of Zoology, University of Oxford, Oxford, United Kingdom; 9 Fogarty International Center, National Institutes of Health, Bethesda, Maryland, United States of America; University of Liverpool, United Kingdom

## Abstract

Livestock contributes directly to the livelihoods and food security of almost a billion people and affects the diet and health of many more. With estimated standing populations of 1.43 billion cattle, 1.87 billion sheep and goats, 0.98 billion pigs, and 19.60 billion chickens, reliable and accessible information on the distribution and abundance of livestock is needed for a many reasons. These include analyses of the social and economic aspects of the livestock sector; the environmental impacts of livestock such as the production and management of waste, greenhouse gas emissions and livestock-related land-use change; and large-scale public health and epidemiological investigations. The Gridded Livestock of the World (GLW) database, produced in 2007, provided modelled livestock densities of the world, adjusted to match official (FAOSTAT) national estimates for the reference year 2005, at a spatial resolution of 3 minutes of arc (about 5×5 km at the equator). Recent methodological improvements have significantly enhanced these distributions: more up-to date and detailed sub-national livestock statistics have been collected; a new, higher resolution set of predictor variables is used; and the analytical procedure has been revised and extended to include a more systematic assessment of model accuracy and the representation of uncertainties associated with the predictions. This paper describes the current approach in detail and presents new global distribution maps at 1 km resolution for cattle, pigs and chickens, and a partial distribution map for ducks. These digital layers are made publically available via the Livestock Geo-Wiki (http://www.livestock.geo-wiki.org), as will be the maps of other livestock types as they are produced.

## Introduction

Livestock contributes directly to the livelihoods and food security of almost a billion people [1]–[2]. In recent decades, the world food economy has seen a shift towards increased consumption of animal-source foods. In developing countries, the meat and dairy sectors have grown at average yearly rates of 5.1 percent and 3.6 percent respectively since 1970 [3]. Most of this increase has been to supply expanding urban areas and has been primarily driven by population growth and increasing incomes, closely linked to urbanisation, making livestock one of the fastest-growing sectors in agriculture [1]. Globally, as human population growth slows and relatively high consumption levels of animal-source foods are reached in some of the countries that have seen the more rapid increases, Brazil and China in particular, growth in demand for meat, milk and eggs is also expected to slow down [4]. Notwithstanding regional reductions in growth, in many developing countries *per capita* consumption of animal-source foods is projected to continue rising and even to accelerate [3]. As well as the economic benefits that a growing livestock sector confers on the economies of these countries, and the potential improvements in food security and nutrition among the world's poor, some 766 million poor (<US$ 2 per day) livestock keepers could benefit directly [5]. This is particularly the case in mixed farming systems where livestock serve many socio-economic functions and promote arable agriculture through manure and draft power [6].

Much of this demand-led growth, and particularly that stemming from the burgeoning urban centres, is likely to be supplied by intensification of production and will generally not be met by small-holders except in particular circumstances where small-holders are well-connected to markets and do not suffer from poor economies of scale, such as the small-holder dairy sector in India [7]. Whilst livestock sector growth presents economic opportunities, in cases where competition from integrated, industrial producers and increasingly tight sanitary standards marginalise the livestock-dependent poor and other small-scale farmers by forcing them out of markets [4], [8]–[9], its main social effects on low-income populations may be negative if not carefully managed.

The growing livestock sector also places increased pressure on natural resources and the environment; making a significant contribution to global environmental change [10]–[11]. Also of concern are the public health implications of livestock production and intensification. As pressure on our global resources increases, people and their livestock are pushed into ever-closer proximity with natural areas and the habitats of wild fauna [12]. This increases the chances of emergence and spread, in livestock and people, of infectious zoonotic pathogens originating in wild animals [13]–[15].

To address these and other such issues requires accurate and accessible and information on the distribution and abundance of livestock. Analyses based on reliable livestock sector data can then be used for targeting and impact assessment, epidemiological evaluation, and to inform planning and policy formulation to promote safe, sustainable and equitable livestock sector development.

Until now, the only global estimates mapped at sub-national resolution have been the 2007 Gridded Livestock of the World (GLW) [16], though the distributions of some species have been modelled over more restricted areas. Neumann and colleagues, for example, modelled and validated the distribution of livestock in Europe by downscaling harmonised livestock statistics through a series of explanatory factors [17]. Other studies have modelled the distribution of poultry species in China and of ducks in Monsoon Asia [18]–[19].

The GLW provided modelled livestock densities of the world at a spatial resolution of 3 minutes of arc (about 5×5 km at the equator). The estimated densities were based on statistical relationships between observed densities within administrative units (‘training data’) derived from survey and census data, and several explanatory variables, including a time-series of remotely-sensed satellite data relating to climate and the environment, and other spatial data relating to demography, land cover and terrain. Reported sub-national statistics were thus spatially disaggregated and gaps where none were available filled with predictions to provide a complete global distribution map for each species.

The GLW maps have been put to a wide variety of uses. They were the fundamental inputs for mapping current and projected estimates of consumption and production of animal-source foods [7]. They have been applied to the analysis of land use dynamics [20] and of global ecosystem services [21] and have contributed to the estimates of the environmental impacts of livestock [22]–[24] and to the characterization of livestock production systems in Eastern Africa [25]. The GLW data have been used in several epidemiological and ecological studies of livestock disease distributions, including trypanosomosis [26]–[27], highly pathogenic avian influenza [28]–[29] and foot-and-mouth disease [30]. The livestock densities have been combined with information on livestock production systems and on production parameters, to estimate the impacts livestock disease and the economic benefits of removing these constraints to production. Examples from cattle in Africa include brucellosis [31] and trypanosomosis [32]–[33].

Since the GLW datasets were first published, considerable advances have been made to improve a) the sub-national statistics on which livestock distributions are based, b) the predictor variables, masks and stratification layers used in the modelling process, c) the modelling methodology itself, and d) the statistical evaluation of modelled results. Some of initial improvements were described in relation to poultry distributions in Asia [18], [19] and methods have since been further developed. Here, we describe the current revised approach in detail and present new global distribution maps, at 1 km spatial resolution, for cattle, pigs and chickens and a partial duck distribution map.

## Materials and Methods

**Figure 1:**
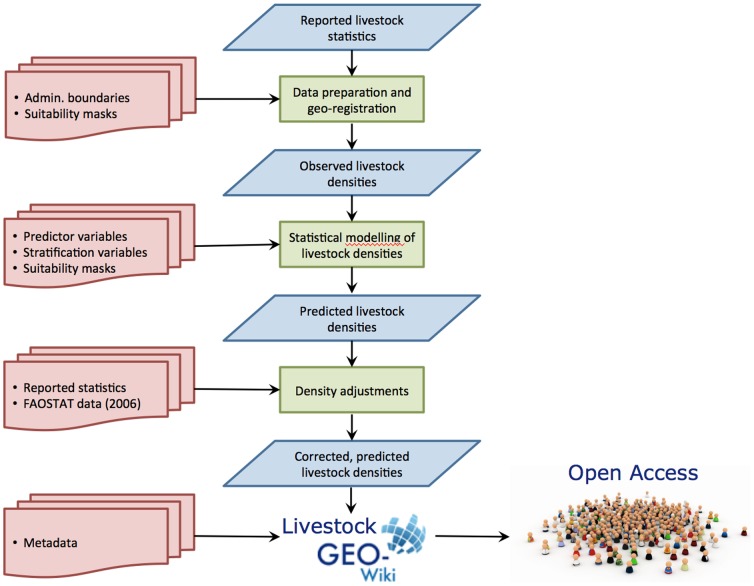
Overall workflow of GLW modelling.

Producing the global livestock distributions for the GLW was a labour-intensive and time-consuming process. Whilst the majority of time is taken in the laborious and painstaking work of acquiring, cleaning and standardising sub-national statistics and registering these to geospatial files of often-changing sub-national boundaries and international borders, it was clear that to become sustainable the data management and livestock mapping processes had to be automated as far as possible. Improvement in data management has been achieved by the development of GLIMS: the Global Livestock Impact Mapping System [34]. Significant advances in livestock distribution modelling have been made possible through the development of a suite of sequentially-implemented R-scripts that produce GLW version 2. The overall workflow from the collection of input data to post-processing and dissemination of outputs is illustrated in Fig. 1.

### GLIMS and sub-national livestock data

Wint and Robinson [16] listed and described the variety of sources for sub-national livestock statistics. These data are collected and cross-referenced against other sources (such as earlier statistics and FAOSTAT national totals). They are then linked to Geographical Information System (GIS) files of the administrative areas at the level to which the livestock statistics are reported. The hierarchical structure and nomenclature of these administrative units varies from country to country but typically includes units such as regions, provinces or districts. This involves a careful process of matching place names, often in different languages, and editing the digital boundaries to account for the merging and splitting of administrative areas, as well as the assignation of livestock numbers to newly defined administrative units. Where possible the FAO Global Administrative Unit Layers (GAUL) files are used as source of national boundary files (http://www.fao.org/geonetwork/srv/en/main.home).

The resulting GIS files of administrative areas and attributed sub-national livestock statistics are then entered into GLIMS. The GLIMS data warehouse, hosted at FAO headquarters in Rome, provides a repository and the tools to edit, explore, search and retrieve the collated data. GLIMS is used to manage a variety of data pertaining to the livestock sector, which are then processed and distributed through various channels and in numerous formats [36]. A module has been specifically developed in GLIMS to extract data for a specified livestock species and aggregation of countries, which are then offered as training data, in the form of a geospatial polygon vector file containing livestock numbers, to the modelling procedures.

### GLW 2: a revised livestock distribution modelling system

The procedure builds on the method developed by Wint and Robinson [16] for disaggregating livestock statistics, based on environmental and other spatial data. Spatially stratified, statistical regression models are developed using data from a series of sample points within each training data polygon and these models are then applied to the entire one-kilometre resolution set of predictor variables in order to estimate livestock densities, disaggregated over a defined study area. For GLW 2, this basic methodology has been revised and improved in a number of ways and was first assessed by Van Boeckel et al. [19] and Prosser et al. [18] in a detailed analysis of its performances for modelling domestic ducks in Asia and poultry in China respectively. Van Boeckel et al. (2011) looked at how downscaling performance was influenced by the aggregation level of input domestic duck data in Thailand (no data for the country, only one value for the country, administrative level 1 data, and administrative level 2 data) and comparing the predictions to actual admin level 3 data. The result was that downscaling based on the method outlined below was giving relatively good results provided that the training data were available at administrative level 1, and were degraded with coarser (national-level data) input data. In a separate study, Prosser et al. (2011) compared land-use based downscaling with the GLW 2 methodology to predict chicken ducks and geese in China, and found land-use based methods to give lower performances. In another previous study, though based on a non-stratified implementation of regression methods, Newmann et al. (2009) found comparable results between land-use and regression based downscaling [17]. In human population mapping, the AfriPop and AsiaPop databases are still largely based on land-use weighting of human population density per land use class [35]–[36], whereas new developments of the WorldPop consortium (www.worldpop.org.uk) are moving toward the use of machine learning methods such as random forest or boosted regression trees (Andy Tatem, comm. pers).

The stratified regressions are repeated a specified number of times using random selections of pixels from which to extract dependent and independent variables (bootstraps). This produces multiple models from which the variability as well as the mean values of the model predictions can be calculated for each pixel.

- The procedures for the statistical transformation of variables and their subsequent selection in the regression models has been revised, better to account for non-linearity and to provide a more flexible process of predictor variable selection.
- A method has been developed to combine the results from a number of stratification schemes; choosing for each location the predictions from the best models for that location. This results in an aggregate, mosaic prediction.
- The entire modelling procedure has been streamlined as a suite of scripts that allow new gridded livestock distributions to be produced relatively quickly using the programming language R [37] when updated input data become available. Automation also facilitates the evaluation of the effects of adjusting model parameters.

Because of the sheer volume of input and covariate data, the modelling must be broken down into continental tiles. Whilst bespoke geographic tiles can be created for specific tasks or projects, there are six continental tiles that are processed independently, and the global dataset is updated every time a new continental tile is processed (see file S1).

### Data layers

The GIS data layers used in the modelling process fall into 3 categories: 1) masks and pixel area files; 2) the predictor covariates; and 3) the stratification layers. All variables are un-projected, using latitude and longitude coordinates and the WGS84 datum. The images have a pixel resolution of 30 minutes of arc (0.00833 degrees) – a nominal pixel resolution of approximately 1 km×1 km at the equator. All imagery is maintained and used as scaled integer data in Idrisi format [38].

Suitability masking is an important step of the modelling process; firstly for adjusting the reported livestock numbers into the densities used as dependent variables in the regression models, and secondly for masking out unsuitable areas with densities of zero in the modelled results. If, for example, an administrative area of 100 km^2^ is reported to contain 100 cattle, but half of its area is deemed unsuitable for raising cattle, the effective density of cattle on ‘usable’ land is not 1 but 2 head km^−2^. Suitability masking in this version is more conservative that it was in the original GLW [16]; areas of forest and desert are no longer explicitly excluded – it is left for the model to determine the suitability of these – so a single mask is used for both ruminant and monogastric species. Land pixels with elevations higher than 4,750 m above sea level or at a slope of gradient higher than 40 percent are deemed unsuitable as well as urban areas and pixels permanently covered with snow or ice. The digital elevation model (DEM) GTOPO30 of the US Geological Survey (USGS) is the source for elevation data and for derived slope information (https://lta.cr.usgs.gov/GTOPO30). The GLC2000 land cover classification developed by the Joint Research Centre (JRC) of the European Commission (EC) is the source for the land cover categories of urban areas and snow and ice [39].

Areas unsuitable for reasons that are not biological or environmental, for example protected areas, are treated differently in the modelling procedure, and are therefore masked separately. A global mask of protected areas is derived from the 2010 version of the World Database on Protected Areas (WDPA)(www.wdpa.org). The International Union for the Conservation of Nature (IUCN) categories Ia and Ib, II, and III were masked as unsuitable as these are characterised by stringent conservation measures and tight regulation of human activity – the encroachment of roaming cattle and other grazing activities is therefore less likely in these than in other areas [40]. Any sample point falling in an area masked for environmental or biological reasons is maintained as a sample point in the model, with a livestock density of zero. If, however a sample point falls in an area deemed unsuitable for legislative reasons, it is dropped from the analysis. The area may, in fact, be quite suitable for livestock, and a zero value for livestock density would mislead the statistical model. A simple land mask is also employed in the modelling process and a file containing the area of each pixel is required for converting livestock numbers into densities, and vice versa. The independent predictor variables offered to the regression analyses are drawn from a spatial data set that allows the modelling process to take advantage of any relationship between livestock densities and climatic, environmental, demographic or topographic factors. The mainstay of these is a Fourier-processed, decadal time series of geo-physical variables derived from Moderate Resolution Imaging Spectroradiometer (MODIS) satellite data (http://modis.gsfc.nasa.gov) from 2001 to 2008. The variables include two vegetation indices, the land surface temperature and the band 3 middle-infra-red, which is particularly suitable for vegetation mapping. Rogers and associates have described the Fourier processing of satellite data in several studies [41]–[44], while a description specific to the MODIS data series is provided by Scharlemann and colleagues [45]. The use of Fourier-processed time-series data is central to the modelling process since the Fourier variables reveal the seasonal characteristics of the environment. Each multi-temporal series is reduced to seven separate, and quite independent, data layers: the mean, and the phases and amplitudes of the annual, biannual and tri-annual cycles of change. Three additional variables combine the amplitude and phase of the three cycles of change and report the contribution of this combination to the total variance in the seasonal phenology. These are further supplemented by the maximum, the minimum and the variance of the satellite-derived geo-physical variables. Two other MODIS-derived products are included as predictor variables: ‘green-up’ and ‘senescence’, derived by Boston University from the NASA MOD12Q2 MODIS tiled phenology layers (http://www.bu.edu/lcsc/research/land-cover-dynamics) [46]. These represent the dates when new green vegetation is first detected at the beginning of a growth cycle, and the onset of senescence at the end of that cycle. Detailed information on the MODIS-derived predictor variables is available on file S2. Two climate-related variables are also used: the length of growing period (LGP), which has been modelled to 1 km spatial resolution [47]–[48] and the annual precipitation data (synoptic period 1950 to 2000) from the WorldClim climate dataset [49]. Since human activities are associated with livestock distributions, two demographic variables are also included. The first is the human population density in 2006, for which the source is the Global Rural-Urban Mapping Project (GRUMP) [50] (http://sedac.ciesin.columbia.edu/data/collection/grump-v1), with country totals adjusted to match the United Nations population values in 2006. The second is the travel time to areas populated by 50,000 people or more [51]. Finally, the GTOPO30 dataset is the source for elevation and slope information [52]. Table 1 provides a list of primary variables used in the analysis.

**Table 1:** Summary of the predictor variables.

Type of variable	Predictor variables	Source
**Vegetation and climate**	14 Fourier-derived variables from MIR*	Scharlemann et al. (2008) [45]
	14 Fourier-derived variables from LSTday and 14 from LSTnight**	
	14 Fourier-derived variables from NDVI*** and 14 from EVI****	
	Green-up (annual cycle 1 and 2)	Zhang et al. (2003) [46]
	Senescence (annual cycle 1 and 2)	
	Length of Growing Period (LGP)	Jones & Thornton (2009) [48]
	Precipitation	Hijmans et al. (2005) [49]
**Topography**	GTOPO30 Elevation	USGS-EROS (1996) [52]
	GTOPO30 Slope	
**Demography**	Human population in 2006	CIESIN et al. (2004)**** [50]
	Travel time to places with >50,000 inhabitants	Nelson (2008) [51]

*Middle Infra-Red;

**Land Surface Temperature;

***Normalized Difference Vegetation Index;

****Enhanced Vegetation Index;

****Country totals adjusted to UN values in 2006 (http://www.un.org/esa/population/).

A given predictor variable may be associated quite differently with livestock densities in different ecological zones. For instance, daytime temperature may have a negative association with livestock densities in an ecological zone describing arid environments, whilst it may have a positive association in temperate regions. In GLW 2 the analyses are therefore spatially stratified using three stratification layers selected to represent different types of spatial zonation. The first of these comprises 25 discrete ecological zones (EZ25) and is the result of an unsupervised classification of some of the Fourier-processed MODIS data, achieved by first reducing the data using a principal component analysis (PCA), followed by clustering using the ADDAPIX programme [53]. The zonation is based on the mean values of the temperature and vegetation variables, as well as elevation and a vegetation seasonality index derived from the Fourier processed time-series of the satellite imagery to distinguish zones in each hemisphere. This is very similar to the stratification scheme that was used to produce the 2007 livestock densities. A second stratification scheme, that has proved very useful, is the map of global livestock production systems (GLPS version 5) [5]. This distinguishes livestock-only, mixed irrigated and mixed rain-fed farming areas, based on land cover, human population densities and data on irrigation. Each of the three categories is then sub-divided, using data on temperature, elevation and LGP, into four sub-categories: hyper-arid; arid and semi-arid; humid and sub-humid; and temperate and tropical highland. A third stratification scheme is provided by a published dataset on the biomes of the world [54]. Olson and associates subdivided the terrestrial world into 867 ecoregions, nested within 14 biomes. The ecoregions were evaluated as a stratification layer but proved too detailed, resulting in many strata failing to contain sufficient sample points to produce models. The biomes, however, have proved to be a useful means of stratification.

### Modelling procedure

The polygon vector data containing the reported livestock numbers derived from GLIMS is combined with the masks and pixel area image and converted to pixel values of animal densities in areas defined as suitable. These observed densities are then converted to log_10_ (n+1) values in order to normalise the distribution of the dependent variable [55]. A set of sample points is randomly selected at a density of 30 per 10,000 km^2^ (though this sometimes needs to be reduced for very large regions), with the constraint that at least one sample point must be selected per input polygon (i.e. if a polygon is smaller than 333 square kilometres). 75% of these points are used to train models and the remaining 25% are used to test their goodness of fit. The number of bootstraps to be used is specified in terms of the number of sample files and the number of point selections within each. Normally, 25 bootstraps are prepared by creating five different files of sample points and, within each, five different selections of the 75% to be used to train the models. Values for the suitability adjusted livestock densities, predictor variables, and stratification layers are extracted for each sample point in each of the five files.

Regressions are then run for each bootstrap, grouping data points according to each stratification scheme and using data points from each stratum of each stratification scheme. A constraint is imposed that a model can only be developed for a stratum if there are at least 30 unique observations for it. Unique, in this context, means from different polygons, each of which represents a single value for the dependent variable (suitability-corrected livestock density). For large polygons, many points may be included in the sample file but all will share the same livestock suitability-corrected density value and are therefore considered a single observation. An un-stratified model is also produced for each continental tile and this is used in areas where there are insufficient data points in any stratum to create a model for it.

Within each stratum a forward stepwise regression is applied. In contrast to standard stepwise regressions though, variables are entered in pairs; each variable being entered simultaneously with its quadratic term. This is intended to account for non-linearity in the relationship between dependent and independent variables. At each step, the pair of variables that best reduces the model Akaike information criterion (AIC) is maintained. In order to avoid over-fitting by including an excessive number of predictor variables, the procedure is halted when the difference in AIC drops below 1 percent in relative terms or when 2 * loglikelihood is below 6 in absolute terms (which corresponds to a p-value of 0.05 of the loglikelihood ratio test for the inclusion of a pair of variables). For the same reason, the maximum number of variables to be entered in a model is estimated by dividing the number of observations by 15. Then, for each bootstrap, the regression models for each stratum (given sufficient points) in each stratification scheme are applied to the predictor variables selected for inclusion in each model. This results in an image of predicted log10 densities for each stratification scheme plus an additional image of predicted log10 densities with no stratification employed.

The next step is to produce a composite of predicted values for each bootstrap by selecting, for each pixel, the prediction resulting from the best performing stratification scheme. Two options are available for choosing the best performing model: the one with the best (highest) R^2^ value or the one with the best (lowest) residual standard error (RSE). The RSE provides information on the accuracy of predictions regardless of the slope of the regression line. In a single model, a poor R^2^ value may be found concomitantly with a very low (good) RSE around a nearly horizontal line, which would in fact correspond to good prediction. An additional image is produced for each bootstrap, which shows, for each pixel, which of the stratification schemes (if any) contributed the predicted livestock density value used in the composite prediction. The correspondence between the observed and predicted livestock densities for the 25% of sample points reserved for testing is then compared for: a) the un-stratified model; b) the composite models based on best R^2^; c) the composite model based on best RSE; and d) the models based on each of the single stratification schemes included. The statistics used for comparison are the simple Pearson correlation coefficient and the root mean square error (RMSE) between the predicted and observed densities. It is normally the case that the best R^2^ or best RSE composite models out-perform all other models (and of these the best RSE model is preferred for the reasons given above). Sometimes, however, a single stratification scheme consistently performs comparably. In such cases, where there is no significant improvement to be had by using a composite model, the predictions from the single stratification are taken; following the principles of parsimony.

Based on either an individual stratification or an aggregate prediction for each bootstrap the predicted (log10) densities are then averaged across the 25 bootstraps to give the mean and standard deviation of modelled predictions. It is this mean value on which the final predicted densities are based, and the standard deviation of this mean shows the consistency in predictions of the bootstraps.

The first step in post-processing is to adjust the predicted densities within polygons for which observations were available such that the predicted totals (of livestock numbers) match those of the reported statistics in the input polygon data (where no observed data are indicated for a polygon by a value of −9,999 the predicted density is retained). In a second post-processing stage the polygon-corrected density maps are further adjusted to match FAO's official national statistics (from FAOSTAT, accessed in 2013), providing a time-standardised datasets for the year 2006. The year 2006 was selected for these livestock distribution maps since it is the base year from which FAO's revised agricultural projections to 2030 and 2050 have been made [3].

Further corrections are made to the predicted densities for pigs, for which a value of zero is applied to all pixels in countries where Islam is the majority religion. Masking only occurs in countries where all of the following conditions are met: 1) more than 50 percent of the population is Muslim; 2) sub-national GLIMS data indicate zero or no data; and 3) FAOSTAT indicates zero or no data for pigs but does report data for other livestock species (see file S3).

### Accuracy of predictions

Beyond the intrinsic ability of the available variables to explain the variance in livestock densities, the goodness of fit of the regression models depends on the level of detail in the input data. Better model performances are expected with more observed data obtained at a lower administrative level [19]. To illustrate this, two tests were undertaken. The first example analysed the predictive accuracy of separate models for cattle in South America by degrading the input data from Brazil to increasingly coarse levels. Recent statistics (2009) are available for Brazil at municipality level (administrative level 2). Brazil accounts for 5,510 out of 7,217 total input polygons in South America so it is expected strongly to influence the continental model for cattle. A similar approach was used in the second test by evaluating the impact of using increasingly coarse training data from Thailand to predict chicken distributions in the country. Sub-district level (locally called *tambon*, corresponding to administrative level 3) statistics on chicken distributions are available for Thailand and, at this level, the country accounts for nearly one third of all the input polygons of the Asia tile. In both examples, the goodness of fit of predicted models was calculated against the input data at the highest administrative level.

## Results

**Figure 2:**
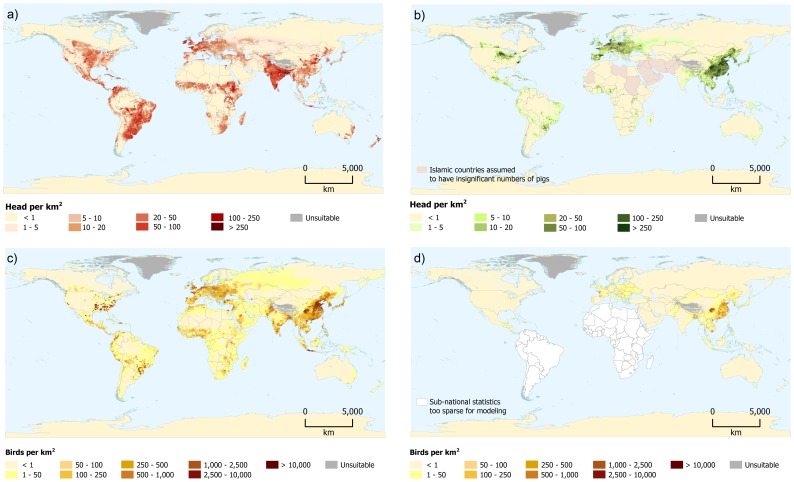
GLW 2 global distributions of a) cattle; b) pigs; c) chickens; and d) distribution of ducks, excluding South America and Africa.

Figure 2 shows the global distributions of cattle, pigs and chickens and the partial distribution of ducks respectively; created by merging the results from the continental tiles for each species. These global maps represent the predicted data, first corrected to match the polygon values of the observed data and then to match the FAOSTAT country values in 2006. The highest cattle densities (Figure 2a) are found in India, in the East African highlands (particularly in Ethiopia), in Northern Europe and in South America. Desert areas and the tropical rain forests of Amazonia and of the Congo Basin have practically no cattle. The highest concentrations of pigs are found in China and in other Eastern Pacific countries (Figure 2b). Pigs are also densely distributed in European countries while only a few countries in Africa (e.g. Uganda, Burkina Faso, Ghana, Nigeria and Togo) have significant densities. Relatively high concentrations are also found in Central America and in Brazil. The distribution of chickens (Figure 2c) closely follows that of the human population. The highest concentrations are found in eastern China, in Pakistan and India, and in western Europe. In Africa, the countries facing the Gulf of Guinea and Madagascar also have high chicken densities. The densely populated east coast of the United States also shows high numbers whilst chickens are only sparsely distributed in the central and western states. The heavily populated areas of southern Brazil also show high concentrations of chickens. The distribution of ducks (Figure 2d), for those regions for which sub-national statistics were available, adds information to previous national and regional duck mapping efforts [18], [19]. Ducks are far less common than chickens worldwide although high densities are to be found in South-East Asia and China where duck production is often integrated with rice cropping and fish farming [56]–[57]. Though to a lesser extent, duck densities are also quite high in a few European countries (e.g. France). file S4 provides a summary of the sub-national statistics used for the modelling and file S5 provides detailed metadata for the sub-national statistics used to develop the livestock distribution models. file S6 provides two graphic summaries of data availability for the modelled species (a) the average spatial resolution of the training data and (b) administrative level of the training data.

**Figure 3:**
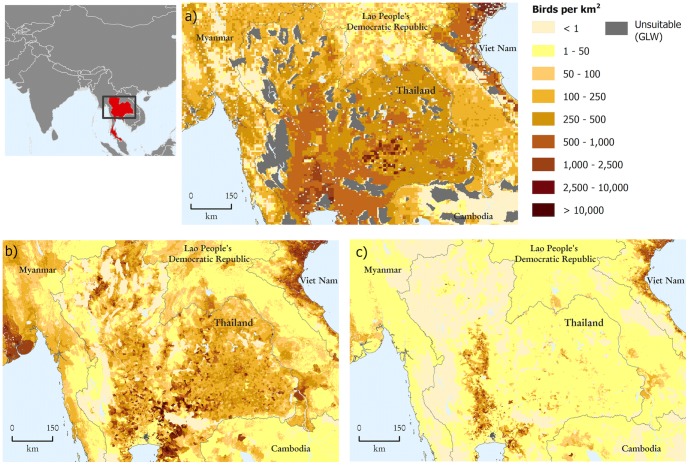
Thailand, visual comparison of a) poultry distribution as mapped in GLW at 5 km; against b) chicken and c) duck distributions mapped separately in GLW 2 at 1 km spatial resolution.

The thematic detail in GLW 2 has been significantly improved compared to the previous version; GLW 2007. Figure 3 zooms in on Thailand and its neighbouring countries and compares the original 2005-corrected distribution of poultry (a) with the new 2006-corrected maps of chickens (b) and ducks (c). Such thematic disaggregation is particularly important in this region because of the abundance of ducks and their important role in farming systems in this part of the world. The figure also illustrates that whilst GLW 2007 explicitly excluded the unsuitable areas based on expert opinion, GLW 2 applies a more conservative approach and leaves to the model to predict the livestock distribution. The two approaches however agree substantially in their mapping of areas with low or zero densities.

**Figure 4:**
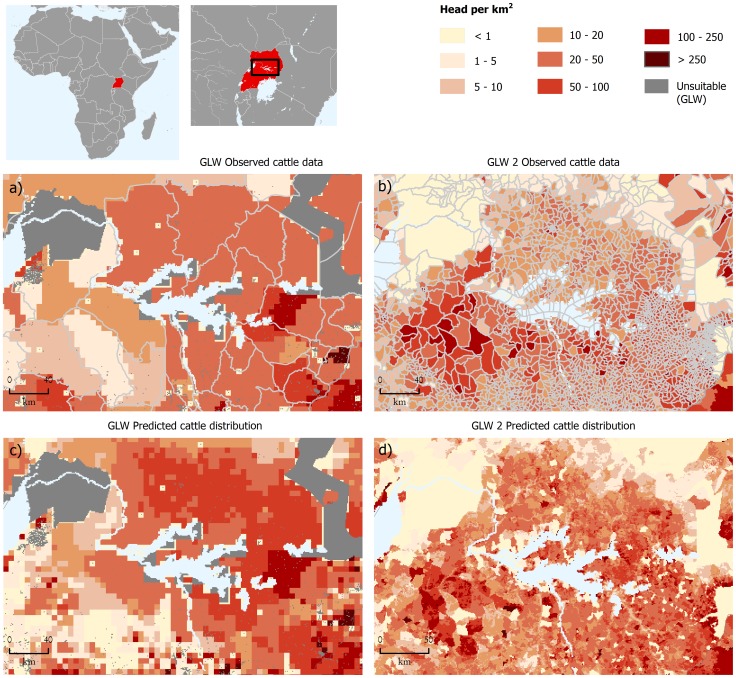
Uganda, visual comparison of observed cattle data a) in GLW 2007 (level 1) and b) in GLW 2 (level 4), and the resulting predicted distribution c) for GLW 2007 and d) for GLW 2.

The finer spatial resolution of the GLW 2 maps (approximate cell size of 1 km^2^ at the equator) also significantly increases the detail of predictions compared to the original version (approximate cell size of 5 km^2^ at the equator). Figure 4 helps to illustrate this with an example from central Uganda. In this case, the finer pixel size combines with a higher disaggregation of the training data; input data were at administrative levels 1 and 4 respectively in the GLW 2007 and GLW 2.

As so very often the case, it is the gathering and processing of primary data – the sub-national statistics on livestock numbers – that is the most arduous and time-consuming part of the process. This operation is necessarily very labour-intensive and usually requires cases-by-case judgements to be made, in particular regarding the linking of reported statistics to geographical units. Few advances could be made, therefore, with respect to earlier approaches. The development of GLIMS, however, has greatly reduced the time and effort needed to store, query and prepare the livestock statistics for modelling. More importantly, the modelling procedure is easily repeated; the suite of R scripts is readily adapted to modelling recurrent updates, different species, different geographical tiles and different technical specifications for statistical modelling and aggregation of results. Automated post-processing activities further allow the rapid production of country-corrected maps for any specified year, global merging of continental tiles, and aggregation to coarser spatial resolutions, as needed.

Computer processing time was primarily a function of the extent of the continental window (i.e. the number of pixels), of the number of polygons contained in the input shape file and of the technical characteristics of the machines used for modelling. Processing time varied from a minimum of 17 hours for the duck model in Europe to a maximum of 210 hours (or 8.7 days) for the cattle model in Asia. file S7 reports the running times for each species and tile, the number of polygons processed in the input training data and the technical specifications of the computers that were used.

Table 2 summarises the fitting metrics for each tile, species and stratification scheme. It reports both the correlation coefficients and the RMSE between observed and predicted values in the validation data sets. The correlation coefficient is an indication of the precision of the predictions, i.e. the extent to which the observed and predicted values are proportional to each other. However, even with a nearly perfect correlation, the predicted values can be wrong in absolute terms if, for example, they systematically overestimate the population. The RMSE, in contrast, is an indicator of the accuracy of the predictions, i.e. how far they are, on average, from the observed values. The highest correlation coefficients between observed and predicted values are typically found for the species and tiles for which observed data are at a higher spatial resolution and are evenly distributed within the modelling window. The models for the Asian tile had the best correlation coefficients compared to models for other continents: pigs and ducks (0.81), chickens (0.74), and cattle (0.63). Better accuracy of predictions was generally found for cattle compared to other species with RMSE values as low as 0.33 and 0.35 in South and North America respectively. RMSE values for cattle and pigs were consistently lower than they were for chickens. Results in Table 2 also indicated, for a given species, that the best stratification differed across the six continental tiles. For the duck models, the biomes stratification scheme gave the lowest RMSE values in the European and North American tiles and performed similarly to the GLPS stratification in Oceania. Composite stratifications had the lowest RMSE values for all species in Asia, but this was the only tile for which the composite prediction performed consistently better than one of the individual stratification schemes. The biomes stratification performed the best in the North American tile, regardless of the species being modelled, and the EZ25 stratification consistently best in South America.

**Table 2:** Summary of the fitting metrics for each continental tile and species modelled.

Tile	Species	Coefficient of correlation	Stratification	RMSE
**Africa**	Cattle	0.63	GLPS	0.42
	Chickens	0.66	GLPS	0.45
	Pigs	0.60	EZ25	0.40
	Ducks	n.a.	n.a.	n.a.
**Asia**	Cattle	0.63	Best RSE	0.46
	Chickens	0.74	Best RSE	0.51
	Pigs	0.81	Best RSE	0.45
	Ducks	0.81	Best RSE	0.57
**Europe**	Cattle	0.77	EZ25	0.42
	Chickens	0.42	EZ25	1.00
	Pigs	0.57	GLPS	0.67
	Ducks	0.55	Biomes	0.54
**North America**	Cattle	0.63	Biomes	0.35
	Chickens	0.59	Biomes	0.88
	Pigs	0.66	Biomes	0.45
	Ducks	0.57	Biomes	0.22
**Oceania**	Cattle	0.50	Biomes	0.48
	Chickens	0.63	Best RSE	0.59
	Pigs	0.72	Biomes	0.54
	Ducks	0.54	GLPS	0.59
**South America**	Cattle	0.73	EZ25	0.33
	Chickens	0.56	EZ25	0.77
	Pigs	0.64	EZ25	0.41
	Ducks	n.a.	n.a.	n.a.

**Figure 5:**
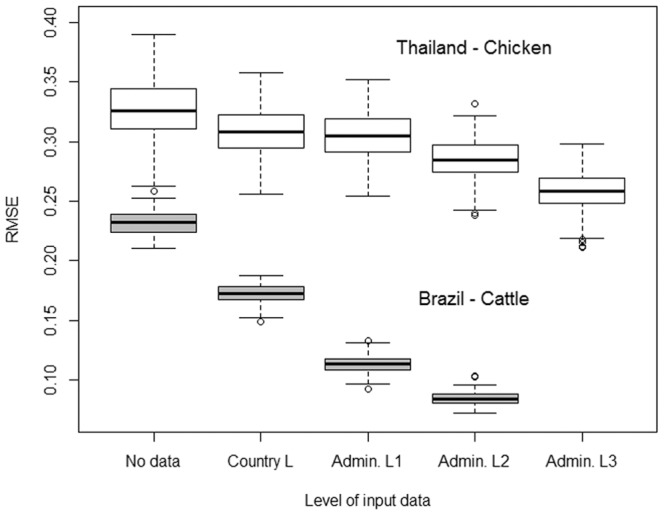
Residual Mean Square Error (RMSE) for predicted versus observed cattle distributions in Brazil, and chicken distributions in Thailand, by administrative level of training data.

The results of the tests carried out to evaluate the influence of the administrative level of input data on the accuracy of the predictions are presented in Figure 5. In both countries and species tested, the accuracy of the predictions decreased (increasing RMSE values) as the administrative level of the input data became coarser. It is notable also that the RMSE values for the cattle predictions in Brazil were lower at all administrative levels than those for the chicken predictions in Thailand.

## Discussion

The new GLW 2 livestock density maps described above provide a timely update of the GLW 2007 livestock distributions [16] and the enhnaced methods and automated procedures mean that updates will, in future, be more frequent than they have been to date. The differences in the modelling procedures, the type and resolution of predictor variables and the different unsuitability masks prevent an explicit, quantitative assessment of change between the livestock densities as mapped by the old and new GLW version. However, we can discuss the improvements that have been made over the original GLW, the accuracy of the predictions and areas where further advances could be made.

### Improvements

The first and most obvious improvement is that GLW 2 provides more contemporary data on the distribution of livestock. The original GLW was published in 2007, but many of the reported statistics on which the predictions were based dated back to the 1990's. Whilst some data in the current set of reported statistics remain relatively old, especially in poor countries where censuses have not been carried out for many years, the median year for which livestock statistics have been obtained is much more recent, with most input data dating being more recent than 2000 (see file S4).

The second important improvement is in the spatial resolution of the training data, allowing the full potential of the higher spatial resolution (1 km) in the predictor variables to be availed. This is clearly shown in Figures 5, which illustrates how the use of higher resolution training data and predictor variables have allowed the spatial disaggregation of livestock census data at an unprecedented spatial resolution. Whilst the 1 km resolution may not be required for many applications, such as for example risk or impact assessments at global or continental scales, the higher resolution provides more opportunities for analysis and modelling to be carried out at the scale of individual countries; greatly extending the use of GLW 2. In some countries, the level of detail in the input data is so fine as to bring into question the usefulness of further disaggregating livestock densities to pixel-level. The reasons for continuing with the modelling, even for small polygons, are four in number. Firstly, even small administrative units are rarely homogeneous in terms of land use and farming so, provided that the covariates are accurate at the finer resolution, the data can still be spatially disaggregated in a meaningful way. Secondly, the training data come in heterogeneous administrative units, which would be otherwise difficult to compare between countries. Spatial disaggregation to pixel level harmonises the spatial scale of the estimates and thus facilitates comparison. Thirdly, even fine-scale input data often have gaps, caused for example by administrative units where no census data are available or are of restricted access. The modelled distributions allow prediction of livestock densities to be made in those areas. The fourth reason is possibly the most important. Statistical models based on smaller administrative units will capture more of the underlying environmental variability, and are statistically more robust.

A third important improvement in GLW 2 is the estimation of uncertainty around the predicted livestock densities. Whilst the modelling procedure does not provide a full and explicit integration of uncertainty based on a Bayesian framework, the bootstrapping procedure allows coefficients of variation to be calculated around the mean estimates; provides an indicative estimate of the degree of consensus among the different models around the mean estimate.

Finally, the breakdown of species and species groups has also been changed. For example, the previous estimates of poultry densities have been replaced by separate estimates for chickens and ducks. Whilst only cattle, pigs, chicken and ducks have been presented here, GLW 2 is currently being applied to produce distribution maps for other species including goats, sheep and buffaloes, as well as regional maps of camels and equines. This level of species detail is essential for some epidemiological investigations, for example in understanding the geographical distribution of highly pathogenic avian influenza (HPAI) H5N1, where treating chickens and ducks separately has greatly enhanced our ability to predict risk [29].

These new GLW 2 outputs are already finding applications in diverse fields. The new pig distribution maps are currently being applied to a global analysis of current and future pig production, in which the pig distributions are further disaggregated by production system. The latest version (5) of the GLPS [5], which is based on land cover data and agro-ecological conditions, maps potential systems rather than actual livestock systems. Integrating these livestock distribution maps with the GLPS will help bring these estimates closer to reality by showing where livestock actually are, rather than where we think conditions are suitable for them to occur. In combination with the revised agricultural projections to 2030 and 2050, recently released by the Agricultural Development and Economic Division (ESA) of FAO [3], these new maps are proving important inputs for further efforts to map the demand and supply of animal-source foods [7]. Likewise, updated global maps of livestock distributions will continue to support analyses that quantify greenhouse gas (GHG) emissions from both the ruminant and monogastric livestock sectors [58]. Finally, the revised maps will find many important epidemiological applications to study disease risk and estimate the impacts of diseases not only in livestock but also for those zoonotic diseases that spill over into the human population. The poultry distributions have already been incorporated into a number of HPAI H5N1 risk assessments [35], [59], and their importance has been highlighted in assessing the risk of spread of the recently emerged H7N9 virus in China and beyond [60]–[61].

Future updates of GLW 2 to include new data as they become available will be greatly facilitated by the integration of the modelling procedure into a fully scripted workflow. The post-processing is part of this automated workflow and allows a variety of outputs to be derived with relative ease and speed. The reference output presented here is a global map of animal densities at 1 km resolution adjusted at the country level to match FAOSTAT 2006 totals. However, outputs expressed in absolute numbers (rather than densities), aggregated to different spatial resolutions (5 km, 10 km or 20 km), or matching different FAOSTAT country totals can also be derived.

### Prediction accuracy

The accuracy of the predictions varied significantly according to species and the level of detail in the input training data, and were comparable to those found by Van Boeckel et al. (2011) in Asia, Van Boeckel et al. (2012) in Thailand, and Prosser et al. (2011) in Asia. The administrative level of the training data had a strong influence on model accuracy; with greater accuracy observed with higher administrative levels of training data for both cattle in Brazil and chickens in Thailand (Figure 5). The modifiable areal unit problem (MAUP), well known to quantitative geographers [62], has been demonstrated to have varying effects with different levels of aggregation of input data. Even though the absolute levels of accuracy in prediction for chickens in Thailand and cattle in Brazil were quite different, the fact that we found a positive association between model accuracy and the administrative level at which the training data were used suggests that, wherever possible, data should be sought and collected at the highest possible resolution to train models, even if this results in heterogeneity in administrative levels used to train models, a result comparable to the one found by Van Boeckel et al. (2011) for domestic ducks in Thailand.

Another observation is that the predictions in Europe and North America for cattle and ducks, whose distributions are conditional on presence of suitable pasture and access to water, were consistently better (i.e. had lower RMSE values) than those for chickens and pigs, whose production is highly intensive in these parts of the world (Table 2). In contrast, in a continent like Africa, where the majority of chicken and pig production is extensive, the accuracies of the models for cattle, pigs and chicken were comparable. These observations relate to the influence of the intensity of livestock production on the accuracy of the predictions. As production intensifies it becomes increasingly detached from the land resource base (for example as feeds are brought in that are grown in completely different places) and thus more difficult to predict based on spatial, agro-ecological variables. This effect is particularly marked for chickens and pigs, where the locations of intensive farming units often have more to do with accessibility to markets or to inputs of one sort or another, than to the agro-ecological characteristics of the land that can be quantified through remotely sensed variables. In support of this, Van Boeckel and colleagues [63] found significantly lower predictability for models of intensive chicken production than for models of extensive chicken production in Thailand. As chicken production becomes intensified in many countries, chicken distributions will become increasingly difficult to predict geographically using largely agro-environmental predictors. This inverse relationship between predictability and the level of intensification is well illustrated by the case of chickens in Europe (the model which performed least well, with an RMSE value of 1.0). The regression statistics suggest that the environmental predictors can only partially explain the distribution of chickens and that other factors that are not currently included in the regression models, relating to policy and economics, for example, are likely to be relatively more important in determining the distributions of chickens in such settings. With general trends toward intensified production, the modelling could be improved in the future by incorporating a wider set of anthropogenic, socio-economic and perhaps demand- or trade-related predictor variables. For intensive production systems, the intrinsic variability is also expected to be higher. A chicken production unit, falling into a single 1 km^2^ pixel, may contain up to a million birds. The characteristics of that pixel, in all covariates, may be identical to those of a neighbouring pixel where an equivalent production plant was not installed. However, the difference in numbers will be so high that the stochastic decision to place the plant in one pixel rather than the other equivalent one will increase the variability that the model cannot capture, even with the most pertinent covariates.

### Validation

A true validation of the predictive accuracy of these models would involve field observation of livestock densities in different pixels and testing those observations against predicted values. However, livestock census data are generally collected and distributed by area (administrative units) and so validation would have to be done on re-aggregated model predictions. Moreover, such validation would be extremely costly and time-consuming. Aside from artificially degrading the training data contributing to models, as we have done here (Figure 5), to make an internal validation of the disaggregation efficiency, there are few options for validation. One possibility which is being explored is the Livestock Geo-Wiki, currently under development along similar lines to the Geo-Wiki produced by the International Institute for Applied Systems Analysis (IIASA) [64]–[65]. Geo-Wiki uses Internet crowd-sourcing to validate and modify land cover information. It is a web-based application with Google Earth as a backdrop, over which various global land cover datasets are draped. Discrepancies in land cover assignation among the datasets can be highlighted and values may be either corroborated or changed by users logged onto the wiki. The development of the Livestock Geo-Wiki was proposed by Robinson and colleagues [5] as a way to validate livestock production systems information and it is hoped that some form of validation will also be possible for livestock distributions. The roles of the Livestock Geo-Wiki for the livestock distribution data described here are, however, in viewing and providing open access to the data.

### Dissemination and future developments

An important objective of the Livestock Geo-Wiki is to disseminate the data. Currently, data on livestock production systems and livestock densities are disseminated via the Gridded Livestock of the World (GLW) website, hosted by FAO (http://www.fao.org/ag/againfo/resources/en/glw/home.html). Whilst this has provided a valuable information resource it is not interactive so the opportunities for users to provide feedback are limited. The Livestock Geo-Wiki has several advantages over more static ways of disseminating data, including, for example: (a) providing a central repository where many different aspects of livestock information may be explored in a highly interactive way; (b) publicising and disseminating open-access livestock sector data at a range of spatial scales; (c) using crowd-sourcing approaches to validate and improve livestock data; and (d) providing innovative data visualisation and analysis tools that will facilitate the investigation of dependencies among the data sets and address specific requirements of diverse groups of users. Instructions on the procedure to download the livestock data from the Livestock Geo-Wiki (http://www.livestock.geo-wiki.org) are provided in file S8.

The improvements to global livestock distribution data, presented here, have been motivated by the pressing need for higher resolution and more contemporary outputs than provided by the original GLW 2007 livestock distributions. Although the methodology has been considerably revised in many aspects, it remains similar to that developed by Wint and Robinson for GLW 2007 [16], involving the use of several stratified multiple regressions linking observed livestock densities to environmental data. New machine-learning methods such as Boosted Regression Trees, or Random Forests have recently demonstrated their increased predictive capacities over more standard statistical methods for species distribution modelling [66]. These methods can better account for non-monotonic relationships between dependent and predictor variables, and can better incorporate the effects of interactions among predictors. Currently, the comparative strength of the stratified regressions lies in the relatively fast fitting of models, and their rapid application to large data sets. However, with the advent of parallelised computing, the use of such machine-learning techniques for modelling livestock distributions is becoming a realistic prospect. These approaches should be investigated in relation to their potential superiority in terms of predictive capacity. In addition, alongside the current set of predictor variables, which are principally environmental in nature, incorporating more socio-economic and anthropogenic predictor variables may confer considerable improvements to models for which the livestock distributions are dominated by more intensified modes of production.

Considerable progress has been made in advancing the spatial modelling of livestock distributions, and there are clearly areas where further improvements can be made. No matter how good the predictive model though, it will always be limited by the quality of the training data on which is based. With this in mind, efforts must be made to identify areas where reported statistics on livestock distributions are out-dated, of low spatial resolution or of doubtful quality, and steps must be taken to address these shortfalls.

## Supporting Information

Information S1 **Specifications for the continental tiles.**
(PDF)

Information S2 **Detailed information on MODIS-derived predictor variables (source, derived Fourier variables, image values and rescaling if applicable).**
(PDF)

Information S3 **Islamic countries in relation to modelling the distributions of pigs.** Countries emboldened in red are those flagged with zero values in modelling the distribution of pigs. (Source: Adapted from Wikipedia http://en.wikipedia.org/wiki/List_of_Muslim-majority_countries).
(PDF)

Information S4 **Aggregate information on sub-national statistics (year and level of observation) by continental tile and species.**
(PDF)

Information S5 5a. Country details on sub-national statistics for cattle (year and level of observation) by continental tile. (file SI5.pdf) Supplementary information 5b. Country details on sub-national statistics for pigs (year and level of observation) by continental tile. (file SI5.pdf) Supplementary information 5c. Country details on sub-national statistics for chickens (year and level of observation) by continental tile. (file SI5.pdf) Supplementary information 5d. Country details on sub-national statistics for ducks (year and level of observation) by continental tile.
(PDF)

Information S6 6a. Average Spatial Resolution (ASR) of the training data used to model a) cattle; b) pigs; c) chickens and d) ducks distributions. The ASR measures the effective resolution of input administrative units in kilometers. It is calculated as the square root of the land area divided by the number of administrative units [1], [2]. (file SI6.pdf) Supplementary information 6b. Country level of detail for the training data of a) cattle; b) pigs; c) chickens and d) ducks.
(PDF)

Information S7 **Polygon details and processing time for each continental tile and species.**
(PDF)

Information S8 **Procedure to download the output raster data.**
(PDF)
