# Supplementary material for: Mapping the Global Distribution of Livestock
Source: PLoS One. 2014 May 29;9(5):e96084. doi: 10.1371/journal.pone.0096084 (PMC4038494; doi:10.1371/journal.pone.0096084)
Supplement: Information S1 — Specifications for the continental tiles. (PDF) [file pone.0096084.s001.pdf]

Supplementary information 1 – Specifications for the continental tiles.

Tile	Xmin	Xmax	Ymin	Ymax	No. rows	No. columns	No. pixels	No. countries
Africa	-18	52	-35	38	8,760	8,400	73,584,000	57
Asia	19	180	-12	85	11,640	19,320	224,884,800	61
Europe	-32	34	34	85	6,120	7,920	48,470,400	42
North America	-180	-50	5	85	9,600	1,560	149,760,000	33
Oceania	94	180	-55	21	9,120	10,320	94,118,400	26*
South America	-95	-30	-60	15	9,000	7,800	70,200,000	16

*Because of the sparse distribution of livestock in the countries of Oceania, the model of this continental tile actually includes 4 Asian countries; allowing for an adequate number of training samples. The predicted values for those Asian countries are however not used.

Country polygons in Africa:

Algeria; Angola; Benin; Botswana; British Indian Ocean Territory; Burkina Faso; Burundi; Cameroon; Cape Verde; Central African Republic; Chad; Comoros; Congo; Côte d'Ivoire; Democratic Republic of the Congo; Djibouti; Egypt; Equatorial Guinea; Eritrea; Ethiopia; Gabon; Gambia; Ghana; Guinea; Guinea-Bissau; Kenya; Lesotho; Liberia; Libya; Madagascar; Malawi; Mali; Mauritania; Mauritius; Mayotte; Morocco; Mozambique; Namibia; Niger; Nigeria; Réunion; Rwanda; Saint Helena; Sao Tome and Principe; Senegal; Seychelles; Sierra Leone; Somalia; South Africa; Sudan; Swaziland; Togo; Tunisia; Uganda; United Republic of Tanzania; Zambia; Zimbabwe.

Country polygons in Asia:

Afghanistan; Armenia; Azerbaijan; Bahrain; Bangladesh; Belarus; Bhutan; Brunei Darussalam; Bulgaria; Cambodia; China; Christmas Island; Cocos (Keeling) Islands; Cyprus; Dem People's Republic of Korea; Georgia; India; Indonesia; Iran (Islamic Republic of); Iraq; Israel; Japan; Jordan; Kazakhstan; Kuril islands; Kuwait; Kyrgyzstan; Lao People's Democratic Republic; Lebanon; Liancourt Rock; Malaysia; Maldives; Mongolia; Myanmar; Nepal; Oman; Pakistan; Paracel Islands; Philippines; Qatar; Republic of Korea; Republic of Moldova; Romania; Russian Federation; Saudi Arabia; Scarborough Reef; Singapore; Senkaku Islands; Spratly Islands; Sri Lanka; Syrian Arab Republic; Tajikistan; Thailand; Timor-Leste; Turkey; Turkmenistan; Ukraine; United Arab Emirates; Uzbekistan; Viet Nam; Yemen.

Country polygons in Europe:

Albania; Andorra; Austria; Belgium; Bosnia and Herzegovina; Croatia; Czech Republic; Denmark; Estonia; Faroe Islands; Finland; France; Germany; Gibraltar; Greece; Holy See; Hungary; Iceland; Ireland; Isle of Man; Italy; Latvia; Liechtenstein; Lithuania; Luxembourg; Malta; Monaco; Montenegro; Netherlands; Norway; Poland; Portugal; San Marino; Serbia; Slovakia; Slovenia; Spain; Svalbard and Jan Mayen Islands; Sweden; Switzerland; The former Yugoslav Republic of Macedonia; United Kingdom.

Country polygons in North America:

Anguilla; Antigua and Barbuda; Bahamas; Barbados; Belize; Bermuda; British Virgin Islands; Canada; Cayman Islands; Costa Rica; Cuba; Dominica; Dominican Republic; El Salvador; Grenada; Guadeloupe; Guatemala; Haiti; Honduras; Jamaica; Martinique; Mexico; Montserrat; Netherlands Antilles; Nicaragua; Panama; Puerto Rico; Saint Kitts and Nevis; Saint Lucia; Saint Vincent and the Grenadines; Turks and Caicos Islands; United States of America; United States Virgin Islands.

Country polygons in Oceania:

American Samoa; Australia; Cook Islands; Fiji; French Polynesia; Guam; Kiribati; Marshall Islands; Micronesia (Federated States of); Nauru; New Caledonia; New Zealand; Niue; Norfolk Island; Northern Mariana Islands; Palau; Papua New Guinea; Pitcairn Islands; Samoa; Solomon Islands; Tokelau; Tonga; Tuvalu; Vanuatu; Wake Island.

Country polygons in South America:

Argentina; Aruba; Bolivia (Plurinational State of); Brazil; Chile; Colombia; Ecuador; Falkland Islands (Malvinas); French Guiana; Guyana; Paraguay; Peru; Suriname; Trinidad and Tobago; Uruguay; Venezuela (Bolivarian Republic of).
