# Supplementary material for: Mapping the Global Distribution of Livestock
Source: PLoS One. 2014 May 29;9(5):e96084. doi: 10.1371/journal.pone.0096084 (PMC4038494; doi:10.1371/journal.pone.0096084)
Supplement: Information S2 — Detailed information on MODIS-derived predictor variables (source, derived Fourier variables, image values and rescaling if applicable). (PDF) [file pone.0096084.s002.pdf]

Supplementary information 2 – Detailed information on MODIS-derived predictor variables (source, derived Fourier variables, image values and rescaling if applicable).

MODIS imagery	Estimated parameter	Number of variables	Estimate Fourier parameter	Number of variables	Image values
MIR	Mean, Minimum, Maximum, Variance,	4	Annual bi-annual, tri-annual amplitude; annual bi-annual, tri-annual phase	6	Reflectance values * 10,000
			Contribution to total variance for combined annual, bi-annual, tri-annual amplitude and phase and total variance of combination	4	
LST (day, night)	Mean, Minimum, Maximum, Variance,	4	Annual bi-annual, tri-annual amplitude; annual bi-annual, tri-annual phase	6	(Degree Centigrade + 273) * 50
			Contribution to total variance for combined annual, bi-annual, tri-annual amplitude and phase and total variance of combination	4	
NDVI and EVI	Mean, Minimum, Maximum	3 + 3	Annual bi-annual, tri-annual amplitude; annual bi-annual, tri-annual phase	6 + 6	Index Value * 1,000
			Contribution to total variance for combined annual, bi-annual, tri-annual amplitude and phase and total variance of combination	4 + 4	
NDVI and EVI	Variance	1 + 1	-------------		Value * 10,000
Green-up	Onset green-up event for annual cycle 1 (1 January - 30 June) and annual cycle 2 (1 July – 31 December)	1 + 1	-------------		Julian day
Senescence	Onset senescence event for annual cycle 1 (1 January - 30 June) and annual cycle 2 (1 July - 31 December)	1 + 1	-------------		Julian day
