# Supplementary material for: Mapping the Global Distribution of Livestock
Source: PLoS One. 2014 May 29;9(5):e96084. doi: 10.1371/journal.pone.0096084 (PMC4038494; doi:10.1371/journal.pone.0096084)
Supplement: Information S3 — Islamic countries in relation to modelling the distributions of pigs. Countries emboldened in red are those flagged with zero values in modelling the distribution of pigs. (Source: Adapted from Wikipedia http://en.wikipedia.org/wiki/List_of_Muslim-majority_countries). (PDF) [file pone.0096084.s003.pdf]

Supplementary information 3 – Islamic countries in relation to modelling the distributions of pigs. Countries emboldened in red are those flagged with zero values in modelling the distribution of pigs. (Source: Adapted from Wikipedia http://en.wikipedia.org/wiki/List_of_Muslim-majority_countries).

Country	Human pop. In thousands	Muslims %	Sunni %	Shia %	Religion & State	Data for pigs		Data for other species
						GLIMS	FAOSTAT*	FAOSTAT*
**Afghanistan**	**28,396**	**99.8**	**85-90**	**10-15**	**Islamic state**	**n.d.**	**n.d.**	**yes**
Albania	3,170	82.1	95	5	None	yes	yes	yes
Algeria	34,895	98.2	99	1	State Religion	yes	yes	yes
Azerbaijan	8,676	98.4	25-35	67-75	Secular	yes	yes	yes
**Bahrain**	**1,047**	**81.2**	**25-35**	**65-75**	**State Religion**	**n.d.**	**n.d.**	**yes**
Bangladesh	142,319	90.4	99	1	State Religion	yes	n.d.	yes
Brunei Darussalam	381	51.9	99	1	State Religion	n.d.	yes	yes
Burkina Faso	13,228	58.9	99	1	Secular	yes	yes	yes
Chad	5,042	55.7	99	1	Secular	yes	yes	yes
**Comoros**	**798**	**98.3**	**99**	**1**	**State Religion**	**n.d.**	**n.d.**	**yes**
Cyprus	288	n.d..	n.d.	n.d.	Secular	yes	yes	yes
**Djibouti**	**496**	**97.0**	**99**	**1**	**Secular**	**n.d.**	**n.d.**	**yes**
Gambia	1,700	95.3	99	1	Secular	yes	yes	yes
Guinea	10,211	84.2	99	1	Secular	yes	yes	yes
Indonesia	228,582	88.1	99	1	None	yes	yes	yes
**Iran (Islamic Republic of)**	**76,923**	**99.6**	**5-10**	**90-95**	**Islamic state**	**n.d.**	**n.d.**	**yes**
**Iraq**	**31,234**	**98.6**	**30-35**	**65-70**	**Secular**	**n.d.**	**n.d.**	**yes**
**Jordan**	**5,569**	**98.8**	**99**	**1**	**State Religion**	**n.d.**	**n.d.**	**yes**
Kazakhstan	16,433	56.4	99	1	Secular	yes	yes	yes
**Kuwait**	**3,400**	**86.4**	**75 - 80**	**20 - 25**	**State Religion**	**n.d.**	**n.d.**	**yes**
Kyrgyzstan	5,357	88.8	99	1	Secular	yes	yes	yes
Lebanon	4,196	59.7	45 - 55	45 - 55	None	n.d.	yes	yes
**Libyan Arab Jamahiriya**	**6,174**	**96.6**	**99**	**1**	**State Religion**	**n.d.**	**n.d.**	**yes**

Malaysia	28,300	61.4	98	2	State Religion	yes	yes	yes
Maldives	350	98.4	99	1	State Religion	n.d.	n.d.	no
Mali	11,995	92.4	99	1	Secular	yes	yes	yes
Mauritania	3,124	99.2	99	1	Islamic state	n.d.	n.d.	yes
Mayotte	194	98.8	99	1	n.d.	n.d.	n.d.	no
Morocco	33,723	99.9	99	1	State Religion	yes	yes	yes
Niger	13,273	98.3	99	1	Secular	yes	yes	yes
Nigeria	155,216	47.9	95	5	Secular	yes	yes	yes
Oman	2,577	87.7	90-95	5-10	State Religion	n.d.	n.d.	yes
Pakistan	172,800	96.4	85-90	10-15	Islamic state	n.d.	n.d.	yes
Qatar	744	77.5	90	10	State Religion	n.d.	n.d.	yes
Saudi Arabia	27,601	97.1	85-90	10-15	Islamic state	n.d.	n.d.	yes
Senegal	11,658	95.9	99	1	Secular	yes	yes	yes
Sierra Leone	6,295	71.5	99	1	None	yes	yes	yes
Somalia	9,559	98.6	99	1	Islamic state	yes	yes	yes
Syrian Arab Republic	22,505	92.8	80-85	15-20	None	n.d.	n.d.	yes
Tajikistan	7,216	99.0	93	7	State Religion	yes	yes	yes
Tunisia	10,384	99.8	99	1	State Religion	n.d.	n.d.	yes
Turkey	73,723	98.6	85-90	10-15	Secular	yes	yes	yes
Turkmenistan	5,433	93.3	33	1	Secular	n.d.	n.d.	yes
United Arab Emirates	5,433	76.0	90	10	State Religion	n.d.	n.d.	yes
Uzbekistan	27,606	96.5	99	1	Secular	yes	yes	yes
West Bank and Gaza Strip	4,169	97.5	99	1	None	n.d.	n.d.	yes
Yemen	23,580	99.0	60-65	35-40	Islamic state	n.d.	n.d.	yes
Sudan	43,940	71.4	99	1	None	0	n.d.	yes
Egypt	79,090	94.7	99	1	State Religion	yes	yes	yes

* FAOSTAT data are taken from the same year from which GLIMS data are available
