# Supplementary material for: Mapping the Global Distribution of Livestock
Source: PLoS One. 2014 May 29;9(5):e96084. doi: 10.1371/journal.pone.0096084 (PMC4038494; doi:10.1371/journal.pone.0096084)
Supplement: Information S4 — Aggregate information on sub-national statistics (year and level of observation) by continental tile and species. (PDF) [file pone.0096084.s004.pdf]

Supplementary information 4 – Aggregate information on sub-national statistics (year and level of observation) by continental tile and species.

Tile	Species	Total Polygons	Years for sub-national statistics (range)	Available level of detail for sub-national statistics				
				Level 0	Level 1	Level 2	Level 3	Other*
**Africa**	Cattle	7,658	1983 - 2011	12	31	17	3	3
	Chickens	6,760	1990 - 2010	25	24	15	1	1
	Pigs	7,992	1990 - 2010	19	23	20	3	1
	Ducks	n.a.	n.a.	n.a.	n.a.	n.a.	n.a.	n.a.
**Asia**	Cattle	19,506	1997 - 2012	17	25	16	5	1
	Chickens	23,870	1996 - 2011	33	15	9	3	5
	Pigs	17,802	2001 - 2011	34	14	11	4	2
	Ducks	23,887	1996 - 2010	43	7	7	3	5
**Europe**	Cattle	13,857	2000 - 2010	11	13	16	2	3
	Chickens	9,741	2000 - 2010	12	15	14	1	3
	Pigs	9,981	2000 - 2010	11	12	17	1	4
	Ducks	636	2000 - 2010	11	12	17	1	4
**North America**	Cattle	6,902	1993 - 2009	15	10	8	---	1
	Chickens	6,827	2000 - 2009	21	4	8	---	1
	Pigs	6,839	2000 - 2009	18	7	8	---	1
	Ducks	6,308	2000 - 2009	18	7	8	---	1
**Oceania**	Cattle	1,744	1989 - 2010	21	5	---	---	1
	Chickens	1,747	2001 - 2007	21	5	---	---	1
	Pigs	275	2001 - 2010	21	4	1	---	1
	Ducks	1,760	2001 - 2009	21	3	2	---	1
**South America**	Cattle	7,217	1994 - 2010	3	6	5	1	1
	Chickens	6,914	1994 - 2009	4	7	4	---	1
	Pigs	7,217	1994 - 2010	2	6	4	1	3
	Ducks	n.a.	n.a.	n.a.	n.a.	n.a.	n.a.	n.a.

*This may refer to sub-national statistics at a higher level of disaggregation than level 3 or sub-national statistics associated with administrative boundaries other than the GAUL. In the latter case, the boundary files are edited to ensure correspondence of national boundaries to those in GAUL.
