# Supplementary material for: Mapping the Global Distribution of Livestock
Source: PLoS One. 2014 May 29;9(5):e96084. doi: 10.1371/journal.pone.0096084 (PMC4038494; doi:10.1371/journal.pone.0096084)
Supplement: Information S5 — 5a. Country details on sub-national statistics for cattle (year and level of observation) by continental tile. (file SI5.pdf) Supplementary information 5b. Country details on sub-national statistics for pigs (year and level of observation) by continental tile. (file SI5.pdf) Supplementary information 5c. Country details on sub-national statistics for chickens (year and level of observation) by continental tile. (file SI5.pdf) Supplementary information 5d. Country details on sub-national statistics for ducks (year and level of observation) by continental tile. (PDF) [file pone.0096084.s005.pdf]

Supplementary information 5a – Country details on sub-national statistics for cattle (year and level of observation) by continental tile.

Africa Tile

COUNTRY	YEAR	LEVEL	Totals in GLIMS	SOURCE
Algeria	2002	1	1,345,593	Direction des Services Vétérinaires - Ministère de l'Agriculture et du Développement Rural 2003 (GLIMS data warehouse)
Angola	2011	1	4,586,569	CountrySTAT - Food and agriculture data network: Número de animais vivos by Ano, Especie, Nivel administrativo. Online data: http://countrystat.org/home.aspx?c=AGO&ta=007SPD035&tr=21
Benin	2011	2	2,058,000	CountrySTAT - Food and agriculture data network: Élevage - Total des effectifs d'animaux vivants 2010 -2012. Online data: http://www.countrystat.org/home.aspx?c=BEN&ta=053SPD135&tr=21
Botswana	2010	2	2,249,364	Central Statistics Office of Botswana: 2009 and 2010 Annual Agricultural Surveys (GLIMS data warehouse)
Burkina Faso	2009	2	8,233,900	Ministère de l'économie et de développement, Direction General des Prévisions et des Statistiques Agricoles: Les régions en chiffres, 2008 & 2010 (GLIMS data warehouse)
Burundi	2005	2	364,732	Direction Provinciale de l'Agriculture et de l'Élevage, Burundi (DPEA): Monographies des les Communes 2006 (GLIMS data warehouse)
Cameroon	2007	1	1,253,931	CountrySTAT - Food and agriculture data network (GLIMS data warehouse)
Cape Verde	2004	2	22,306	Directorate of livestock, Cape Verde: Recenseamento Geral da Agricultura, 2004 (GLIMS data warehouse)
Central African Republic	1990	1	2,501,269	Environmental Research Group Oxford, ERGO: FAO, 2004. Agro-ecological distributions for Africa (Data CD) (GLIMS data warehouse)
Chad	2007	1	7,688,188	Direction de l'Élevage et des Ressources Animales: Effectifs du cheptel national par région administrative 2007 (GLIMS data warehouse)
Comoros	2004	1	37,200	FAO animal production and health division: Rapport National Sur l'Etat des Ressources Génétiques Animales (GLIMS data warehouse)
Congo	1996	1	72,100	Centre National de la Statistique et des Études Économiques, Congo: Estimations des comptes nationaux de 1997-2000 (GLIMS data warehouse)
Côte d'Ivoire	2001	1	1,135,196	Ministère de l'Agriculture et des Ressources Animales, Direction de la Programmation, Côte d'Ivoire: Recensement National de l'Agriculture 2001 (GLIMS data warehouse)
Democratic Republic of the Congo	2004	1	2,899	Centre for Ticks and Tick-Borne Diseases: Preliminary census data from SADC and Eastern Africa (GLIMS data warehouse)
Djibouti	1998	1	297,001	Ministry of Agriculture of Djibouti through IGAD secretariat staff, data for 1998 (GLIMS data warehouse)
Egypt	2011	1	4,779,743	Central Agency for Public Mobilization and Statistics, Egypt: Estimated numbers of livestock and animals by Governorate 2011 (GLIMS data warehouse)

Equatorial Guinea	1985	6	320	International Programme for the Development of the Meat Sector and International Programme for Coordinating the Development of the Dairy Sector. Republic of Equatorial Guinea, Draft Report, FAO, Rome, Italy.
Eritrea	2002	2	1,927,137	Environmental Research Group Oxford, ERGO: GIEWS special reports. Online data: http://www.fao.org/docrep/005/y7678e/y7678e00.htm
Ethiopia	2002	3	45,758,978	Central Statistical Authority of Ethiopia: Agricultural Census 2001-2002 (GLIMS data warehouse)
Gabon	1998	1	18,321	Environmental Research Group Oxford, ERGO: Agro-ecological distributions for Africa (GLIMS data warehouse)
Gambia	2001	2	323,167	Agricultural Statistics and Resources Economics Unit (ASRE), Government of the Gambia: Report of the Agricultural Census of The Gambia 2001/2002 Volume 1 and Volume 2 (GLIMS data warehouse)
Ghana	1996	1	1,247,861	Veterinary Services of Ghana: Livestock Census, 1994-1996 (GLIMS data warehouse)
Guinea	2011	1	5,234,783	Réseau des Systèmes d'Information des Marchés en Afrique de l'Ouest: Stats cheptel. Online data: http://www.resimao.org/html/fr/Guinee/stats#cheptel
Guinea-Bissau	2009	1	1,121,555	Ministère de l'Agriculture et du Développement Rural, Guinée-Bissau: Synthèse des résultats du Recensement National de l' Elevage en Guinée-Bissau, 2009 (GLIMS data warehouse)
Kenya	2005	3	10,714,381	Ministry of Livestock Development, Kenya: Compilation of divisional data 2005 (GLIMS data warehouse)
Lesotho	2010	1	626,344	Bureau of Statistics of Lesotho: 2009/10 Lesotho Agricultural Census, Livestock Report (GLIMS data warehouse)
Liberia	1983	1	12,598	Ministry of Agriculture Regional Livestock Officers, Liberia: Estimates On Livestock Population in Liberia (GLIMS data warehouse)
Madagascar	2005	3	9,500,138	Ministère de l'Agriculture, Secrétariat General, Direction de la Prévision et des Etudes Economiques, Madagascar: Recensement de l'agriculture (RA) - Campagne agricole 2004-2005 (GLIMS data warehouse)
Malawi	2007	2	884,132	Agriculture Statistics Division of the National Statistical Office of Malawi (NSO): National Census of Agriculture and Livestock (October 2006 - October 2007) (GLIMS data warehouse)
Mali	2011	1	9,438,181	CountrySTAT - Food and agriculture data network: Élevage - Total des effectifs d'animaux vivants. Online data: http://www.countrystat.org/home.aspx?c=MLI&tr=21
Mauritania	2007	1	795,500	Office of National Statistics: Monographies Des Régions (GLIMS data warehouse)
Mauritius	2011	1	5,365	Central Statistic Office of Mauritius: Digest of Agricultural Statistics 2011 (GLIMS data warehouse)
Mayotte	2010	1	17,150	Agreste la Statistique Agricole, Le Ministère de l'Agriculture, de l'Alimentation, de la Pêche et de la Ruralité, France: Recensement Agricole 2010. Online data: http://agriculture.gouv.fr/recensement-agricole-2010
Morocco	2008	2	2,651,200	Ministry of Agriculture, Rural Development and Sea Fisheries: Maroc Régions 2008 (GLIMS data warehouse)
Mozambique	2002	2	590,363	Direccao Nacional De Pecuaria, Mozambique: Annual Reports, 2003 (GLIMS data warehouse)
Namibia	2006	1	2,388,780	FAO Special Report, FAO/WFP Crop, Livestock and Food Security Assessment Mission to Namibia (GLIMS data warehouse)
Niger	2005	2	7,336,087	Ministère des Ressources Animales, Niger: Recensement Général de l'Agriculture et du Cheptel. Volume II. Résultats Définitifs (Volet cheptel). Juin 2007. (Projet GCP/NER/041/EC). (GLIMS data warehouse)
Nigeria	2007	1	25,019,700	National Bureau of Statistics, Nigeria: Agricultural Census 2007 (GLIMS data warehouse)

Rwanda	2008	2	1,548,970	National Institute of Statistics of Rwanda: National Agricultural Survey 2008 (Final report) (GLIMS data warehouse)
Réunion	2010	2	16,316	Agreste la Statistique Agricole, Le Ministère de l'Agriculture, de l'Alimentation, de la Pêche et de la Ruralité, France: Recensement Agricole 2010. Online data: http://agriculture.gouv.fr/recensement-agricole-2010
Saint Helena	2009	1	598	Agriculture and Natural Resources Department of St. Helena: Livestock Census 2009 (GLIMS data warehouse)
Senegal	2009	2	3,260,867	Agence Nationale de la Statistique et de la Démographie du Sénégal: Live Data Base (LDB). Online data: http://www.ansd.sn/publications_SES_region.html
Sierra Leone	2004	2	98,632	Ministry of Agriculture & Food Security of Sierra Leone: 2004 Populations and Housing census Statistical Office (GLIMS data warehouse)
Somalia	1996	6	4,409,430	Food Security Assessment Unit (FSAU) in 1999: Livestock Populations in Various Zones of Somalia (FSAU data) (GLIMS data warehouse)
South Africa	2011	1	13,871,000	Ministry of Agriculture, Forestry and Fisheries of South Africa: Trends in the Agricultural Sector, 2011 (GLIMS data warehouse)
Sudan	2009	1	41,563,002	Ministry of Animal Resources and fisheries, Sudan: Statistical Year Book for the Year 2009 (GLIMS data warehouse)
Swaziland	2010	1	619,753	Directorate of Veterinary and Livestock Services: Swaziland Livestock Statistics, Census 2010 (GLIMS data warehouse)
Togo	1995	1	201,078	Data from Dr. Guy Hendrickx: Round Survey, 1995 (GLIMS data warehouse)
Tunisia	2010	1	670,980	Ministère de l'Agriculture de l'Environnement et des Ressources Hydrauliques, Tunisia: Annuaire Statistique 2010 (GLIMS data warehouse)
Uganda	2008	4	10,016,659	Uganda Bureau of Statistics: Uganda census 2008 - Data acquired from Dr. W.Wint (GLIMS data warehouse)
United Republic of Tanzania	2009	2	18,713,654	CountrySTAT - Food and agriculture data network (GLIMS data warehouse)
Western Sahara	2009	1	1,300	Direction de la Statistique du Maroc: Annuaires Statistiques des Régions (GLIMS data warehouse)
Zambia	2009	1	3,445,630	Department of Research and Specialist Services, Zambia: National Livestock Census by Province 1994 to 2009 (GLIMS data warehouse)
Zimbabwe	2008	2	5,106,673	Department of Livestock and Veterinary Services: Livestock Census Summary (GLIMS data warehouse)

No Data for: Bassas da India, British Indian Ocean Territory, Europa Island, Glorioso Island, Juan de Nova Island, Libya, Sao Tome and Principe, Seychelles, Tromelin Island

Asia Tile

COUNTRY	YEAR	LEVEL	Totals in GLIMS	SOURCE
Afghanistan	2003	2	3,706,186	Ministry of Agriculture, Irrigation and Livestock of Afghanistan: Afghanistan - National Livestock Census 2002-2003 - Final Report. Online data: http://reliefweb.int/sites/reliefweb.int/files/resources/58C374339FC582F149256DF30009D60F-fao-afg-4dec.pdf
Armenia	2012	1	599,243	National statistical service of the republic of Armenia: Livestock Census, 2012. Online data: http://www.armstat.am/file/article/anas_2012.pdf
Azerbaijan	2011	1	2,412,305	The State Statistical Committee of the Republic of Azerbaijan: The data on regions. Online data: http://www.stat.gov.az/source/agriculture/indexen.php
Bangladesh	2008	3	28,423,749	Bangladesh Bureau of Statistics: Agriculture Census Report 2008. Online data: http://www.bbs.gov.bd/PageWebMenuContent.aspx?MenuKey=224
Belarus	2011	2	3,930,200	National Statistical Committee of the Republic of Belarus: Agriculture of the Republic of Belarus, 2011 (GLIMS data warehouse)
Bhutan	2009	2	303,018	Ministry of agriculture and forest, Royal government of Bhutan: Dzongkhag livestock sector (GLIMS data warehouse)
Bulgaria	2010	2	572,710	Ministry of Agriculture and Forestry - Agrostatistics Directorate, Bulgaria: Agricultural Census 2010. Online data: http://www.mzh.government.bg/MZH/ShortLinks/SelskaPolitika/Agrostatistics/Structure_agricultural_holdings/Results.aspx
Cambodia	2011	2	3,339,623	Ministry of Agriculture, Forestry and Fisheries: Cambodia Livestock Census 2011 (GLIMS data warehouse)
China	2001	3	135,686,083	China Agricultural Press, 2002. For Taiwan: Statistics Office, COA, Executive Yuan, Taiwan Statistical Yearbook, 2010 (GLIMS data warehouse)
Cyprus	2003	1	61,053	Statistical Service of Cyprus: Census of Agriculture, 2003. Online data: http://www.mof.gov.cy/mof/cystat/statistics.nsf/All/DE20AEA98C4DD6EEC22577A50041F0B8/$file/Census_of_Agriculture-2003.pdf?OpenElement
Georgia	2004	2	1,157,781	National Statistics Office of Georgia: First National Agricultural Census 2004 in Georgia. Online data: http://geostat.ge/cms/site_images/_files/english/agriculture/census/section7.pdf
India	2007	2	186,515,966	Department of Animal Husbandry, Dairying & Fisheries, Ministry of Agriculture, India: Census of Agriculture, 2007 (GLIMS data warehouse)
Indonesia	2008	1	12,714,181	Department Pertanian Republik Indonesia: Buku Statistik, 2009 (GLIMS data warehouse)
Iran, Islamic Republic of	2005	1	7,609,359	Statistical Centre of Iran: Iran Statistical Yearbook 1385 (2005) (GLIMS data warehouse)

Iraq	2000	1	511,644	FAO, 2003. Investment Centre Division Asia and Pacific - Service emergency operation and rehabilitation division - Special emergency programme service data for the three northern governorates. Iraq - SCR 986- three-year agricultural programme in the Iraqi Northern (GLIMS data warehouse)
Israel	2002	1	177,194	Central Bureau of Statistics, Agriculture Division: Annual tables from publication: agriculture in Israel 2001-2003. Online data: http://www.cbs.gov.il/haklaut/agrical_2003/haklaut2003_e.pdf
Japan	2011	1	4,230,570	Statistics Department, Minister's Secretariat, Ministry of Agriculture, Forestry and Fisheries: The 87th Statistical Yearbook of Ministry of Agriculture Forestry and Fisheries, Japan 2011-2012. Online data: http://www.maff.go.jp/e/tokei/kikaku/nenji_e/87nenji/index.html
Jordan	2007	2	88,215	Department of Statistics, Jordan: The Agricultural Census 2007 (GLIMS data warehouse)
Kazakhstan	2012	1	5,690,000	Agency on Statistics of the Republic of Kazakhstan: Basic indicators for 2003-2012. Online data: http://www.eng.stat.kz/digital/Agriculture/Pages/default.aspx
Kuwait	2009	1	35,705	Central Statistical Bureau, State of Kuwait: Annual Agricultural Statistics 2009-2010. Online data: http://www.csb.gov.kw/Socan_Statistic_EN.aspx?ID=42
Kyrgyzstan	2012	1	1,357,015	National Statistic Committee of Kyrgyzstan (GLIMS data warehouse)
Lao People's Democratic Republic	2011	2	1,583,334	Department of Livestock and Fisheries, Laos: Lao Livestock Census Data 2011/12 in: PDR Farm Animal Distributions, 2011: Comparative Maps of Agricultural Census and Administrative Returns (GLIMS data warehouse)
Lebanon	1997	2	56,626	United Nations, Economic and Social Commission for Western Asia, 1999: National farm data handbook for Lebanon. New York (GLIMS data warehouse)
Malaysia	2011	1	768,403	Department of Veterinary Service, Malaysia: Selected Agricultural Indicators 2012 (Indikator Pertanian Terpilih 2012). Online data: http://www.statistics.gov.my/portal/download_Agriculture/files/Selected_Agricultural_Indicators_Malaysia_2012.pdf
Mongolia	2012	2	2,583,027	National Statistical Office of Mongolia (MONSIS): Number of livestock by type, by regions, soums, aimags and the Capital. Online data: http://www.1212.mn/en/contents/stats/contents_stat_fld_tree_html.jsp
Myanmar	2011	3	13,981,114	Livestock Breeding and Veterinary Department (LBVD), Ministry of Livestock Breeding and Fisheries, Yangon, Myanmar: Myanmar Farm Animal Resources: Numbers and Distributions from Administrative Returns, 2011/12 (GLIMS data warehouse)
Nepal	2009	3	7,106,976	Ministry of Agriculture and Cooperatives (MOAC), Nepal: 2009 data (GLIMS data warehouse)
Pakistan	2010	2	24,114,455	Agricultural Census Organization, Ministry of Food & Agriculture, Pakistan: Agricultural Census 2010. Online data: http://www.pbs.gov.pk/content/agricultural-census-2010-pakistan-report
Philippines	2010	3	2,599,466	Bureau of Agricultural Statistics (BAS): Livestock Survey Philippines 2010 (GLIMS data warehouse)
Qatar	2001	1	9,671	Ministry of Municipal Affairs and Agriculture Department of Agriculture and Water Research: Agricultural Census 2000-2001 (GLIMS data warehouse)

Republic of Korea	2011	1	3,353,353	Korea National Statistical Office: Korea Statistical Yearbook, 2012 (GLIMS data warehouse)
Republic of Moldova	2011	2	209,900	Statistica Moldovei: Statistical Yearbook of the Republic of Moldova 2011. Online data: http://www.statistica.md/pageview.php?l=en&idc=263&id=2193
Romania	2011	1	1,988,939	National Institute of Statistics, Romania: Tempo online database/Agriculture/Livestock and Animal Production 2000-2011. Online data: https://statistici.insse.ro/shop/?lang=en
Russian Federation	2012	1	21,446,360	Russian Federation - Federal State Statistical Service: Livestock and poultry production, 2011 and 2012. Online data: http://www.fedstat.ru/indicators/start.do
Saudi Arabia	2009	1	424,489	Central Department of Statistics and Information of Kingdom of Saudi Arabia: Statistical Yearbook, 2010 Saudi Arabia (GLIMS data warehouse)
Sri Lanka	2012	2	1,235,535	Agriculture and Environment Statistics Division, Department of Census and Statistics, Sri Lanka: Livestock Population by Type and by District 2003 - 2012. Online data: http://www.statistics.gov.lk/agriculture/Livestock/LivestockPopulationSubnational.html
Syrian Arab Republic	2004	1	1,024,120	National Agricultural Policy Center - The Ministry of Agriculture and Agrarian Reform: Syrian agricultural database (SAD) (GLIMS data warehouse)
Tajikistan	2009	2	1,834,727	Agency for Statistics, Tajikistan Republic: Regions of Tajikistan Republic, 2010 (GLIMS data warehouse)
Thailand	2006	1	6,003,883	Regional Data Exchange System on Food and Agricultural Statistics in Asia and Pacific Countries: Thailand sub national Statistics. Online data: http://www.faorap-apcas.org/thailand.html
Turkey	2009	2	10,723,958	Turkish Statistical Institute: Livestock statistics database 2009 (GLIMS data warehouse)
Ukraine	2012	1	4,645,900	State statistics committee Ukraine: Head of livestock and poultry by regions (GLIMS data warehouse)
United Arab Emirates	2000	1	96,050	Ministry of Agriculture and Fisheries: Agricultural Statistical Yearbook 2000. United Arab Emirates (GLIMS data warehouse)
Uzbekistan	2002	1	5,416,100	State Statistics Department of Uzbekistan: Agriculture of Uzbekistan, 2001. Minmakroeconomstat of Republic Uzbekistan, State Statistics Department of Uzbekistan Tashkent, 2002 (GLIMS data warehouse)
Viet Nam	2001	6	3,710,385	General Statistics Office of Vietnam: Agricultural Census database 2001. Online data: http://www.gso.gov.vn/default_en.aspx?tabid=477&idmid=4&ItemID=1824
Yemen	2007	1	1,394,707	Directorate of Agricultural Statistics and Documentation, Ministry of Agriculture and Irrigation, Yemen (GLIMS data warehouse)

No Data for: Bahrain, Brunei Darussalam, Christmas Island, Cocos (Keeling) Islands, Democratic People's Republic of Korea, Kuril Islands, Liancourt Rock, Maldives, Oman, Paracel Islands, Scarborough Reef, Singapore, Senkaku Islands, Spratly Islands, Timor-Leste, Turkmenistan

Europe Tile

COUNTRY	YEAR	LEVEL	Totals in GLIMS	SOURCE
Albania	2010	1	493,000	Institute of Statics, Albania: Agricultural sector, Livestock 2001-2010 (GLIMS data warehouse)
Andorra	2010	1	1,565	Ministry of Economy and Territory - Department of Agriculture, Andorra: Agriculture, Herding, Hunting and Fishing - Livestock by type and parish 2003-2010 (GLIMS data warehouse)
Austria	2010	1	2,013,281	Statistics Austria: Livestock Survey, 2010 (GLIMS data warehouse)
Belgium	2010	3	1,878,963	Directorate - General Statistics Belgium formerly known as NIS-INS: Data 2010 from William Wint, (all acquired by Els Ducheyne at Avia_GIS, Zoersel, Belgium, process by William Wint, ERGO Ltd) (GLIMS data warehouse)
Croatia	2003	1	398,037	Central Bureau of Statistics, Republic of Croatia: Agricultural Census 2003. Online data: http://www.dzs.hr/default_e.htm
Czech Republic	2010	2	1,328,008	Czech Statistical Office: Agrocensus regions: Farm Structure Survey and Survey on Agricultural Production Methods 2010. Online data: http://www.czso.cz/csu/2012edicniplan.nsf/engp/2127-12
Denmark	2010	2	1,571,050	Denmark Statistics: Farms by region and selected farms, crops and livestock. Online data: http://www.statistikbanken.dk/statbank5a/SelectVarVal/Define.asp?Maintable=BDF51&PLanguage=1
Estonia	2001	2	280,731	Statistikaamet Statistical Office of Estonia: Agricultural Census 2001. Online data: http://www.stat.ee/agricultural-census-2001
Finland	2010	6	925,800	The Information Centre of the Ministry of Agriculture and Forestry, Finland: Yearbook of Farm Statistics 2011 (GLIMS data warehouse)
France	2009	2	19,692,468	Service de la Statistique et de la Prospective (SSP): Statistique Agricole Annuelle (GLIMS data warehouse)
Germany	2007	2	12,351,818	Federal Statistical Office, Germany: GENESIS database. Online data: https://www.regionalstatistik.de/genesis/online/online;jsessionid=EE45147898822814978BE734145275C4?operation=sprachwechsel&option=en
Greece	2007	2	731,996	Hellenic Statistic Authority (EL.STAT): Farms and number of animals by type, region and county (GLIMS data warehouse)
Hungary	2010	1	684,000	Hungarian Central Statistical Office: Regional Statistics. Online data: http://www.ksh.hu/agriculture
Iceland	2010	2	73,781	Statistics Iceland: 2010 data (GLIMS data warehouse)
Ireland	2000	6	6,851,919	Central Statistical Office Ireland: Census of Agriculture 2000 (data acquired by Els Ducheyne at Avia_GIS, Zoersel, Belgium, process by William Wint, ERGO Ltd) (GLIMS data warehouse) (GLIMS data warehouse)
Italy	2010	3	5,587,719	Istituto Nazionale di Statistica: Censimento Agricoltura 2010. Online data: http://dati-censimentoagricoltura.istat.it

Latvia	2001	1	364,807	Central Statistical Bureau of Latvia: Results of 2001 Agricultural Census. Online data: http://www.csb.gov.lv/en/dati/agricultural-census-2001-30765.html
Liechtenstein	2009	1	6,078	Amt für Statistik Liechtenstein: Landwirtschaftsstatistik 2009 (GLIMS data warehouse)
Lithuania	2010	2	780,051	Lietuvos Statistikas Departamenta: Number of livestock by administrative territory, 2011 (GLIMS data warehouse)
Luxembourg	2008	2	196,295	Portail des statistiques du Grand-Duché de Luxembourg (STATEC): Recensement Agricole, 2008 (GLIMS data warehouse)
Malta	2010	6	15,688	National Statistics Office of Malta: Census of Agriculture 2010. Online data: http://www.nso.gov.mt/statdoc/document_view.aspx?id=3287&backurl=/themes/theme_page.aspx
Montenegro	2010	1	78,633	Statistical office of Montenegro (MONSTAT): Agriculture Census 2010. Online data: http://www.monstat.org/eng/page.php?id=58&pageid=58
Netherlands	2010	2	3,975,194	Centraal Bureau voor de Statistiek, Netherlands: Agriculture: crops, livestock and land use by general far type, region - 2011 (GLIMS data warehouse)
Norway	2010	1	867,641	Statistics Norway: Agriculture Statistics 2011 (GLIMS data warehouse)
Poland	2009	1	5,700,000	Central Statistical Office, Poland: Statistical Yearbook of Agriculture 2010 (GLIMS data warehouse)
Portugal	2009	2	1,173,975	Instituto Nacional de Estatística - Statistics Portugal: Agricultural Census 2009. Online data: http://ra09.ine.pt/xportal/xmain?xpid=RA2009&xpgid=ra_home
Serbia	2006	1	1,105,988	Statistical Office of Serbia: Municipalities of Serbia 2007 (GLIMS data warehouse)
Slovakia	2009	2	471,965	Statistical Office of the Slovak Republic: Livestock by territory, products and period. Online data: http://px-web.statistics.sk/PXWebSlovak/DATABASE/En/databasetree.asp
Slovenia	2010	2	471,584	Statistical Office of the Republic of Slovenia: Agricultural Census Slovenia 2010. Online data: http://www.stat.si/eng/novica_prikazi.aspx?id=4594
Spain	2010	2	6,075,081	Ministerio de Medio Ambiente y Medio Rural y Marino, Gobierno de España (MARM): Encuestas Ganaderas 2010 (GLIMS data warehouse)
Sweden	2009	1	1,538,281	Statistics Sweden: Yearbook of Agricultural Statistics 2010 (GLIMS data warehouse)
Switzerland	2009	1	1,597,484	Swiss Federal Statistical Office: Recensement des entreprises agricoles,1999-2009 (GLIMS data warehouse)
The former Yugoslav Republic of Macedonia	2006	2	232,526	State Statistic Office of the Republic of Macedonia: Census of Agriculture 2007. Online data: http://www.stat.gov.mk/PrikaziPublikacija_en.aspx?id=51&rbr=205
United Kingdom	2009	2	9,991,910	Office for National Statistics, U.K.: Statistics by Region. Online data: http://www.statistics.gov.uk/hub/regional-statistics/

No Data for: Bosnia and Herzegovina, Faroe Islands, Gibraltar, Guernsey, Holy See, Isle of Man, Jersey, Madeira Islands, Monaco, San Marino, Svalbard and Jan Mayen Islands

North America Tile

COUNTRY	YEAR	LEVEL	Totals in GLIMS	SOURCE
Belize	2003	1	57,800	Ministry Of Agriculture, Fisheries and Cooperatives, Belize: Annual Report 2003. Online data: http://www.agriculture.gov.bz/PDF/Annual_Report_2003.pdf
Canada	2006	2	15,772,904	Statistics Canada: 2006 Census of Agriculture. Online data: http://www.statcan.gc.ca/ca-ra2006/index-eng.htm
Cayman Islands	2009	1	2,231	Economics and Statistics Office - Government of the Cayman Islands: Statistical Compendium 2009 (GLIMS data warehouse)
Costa Rica	2000	1	1,358,209	Ministerio de Agricultura y Ganadería, Costa Rica: Censo Ganadero 2000 (GLIMS data warehouse)
Cuba	2009	1	3,586,303	Oficina Nacional de Estadísticas, República de Cuba: Anuario Estadístico de Cuba 2009 - Edición 2010 (GLIMS data warehouse)
Dominican Republic	1997	1	2,453,276	Oficina Nacional de Estadística, Republica Dominicana: Republica Dominicana en cifras 1997 (GLIMS data warehouse)
El Salvador	2007	2	1,141,323	Ministerio de Economía - El Salvador: IV Censo Agropecuario 2007-2008 (GLIMS data warehouse)
Guadeloupe	2000	1	64,236	Agreste la Statistique Agricole, Le Ministère de l'Agriculture, de l'Alimentation, de la Pêche et de la Ruralité, France: Recensement Agricole 2000. Online data: http://agreste.agriculture.gouv.fr/recensement-agricole-2010/resultats-donnees-chiffrees/
Guatemala	2003	2	1,627,522	Instituto Nacional de Estadística, Guatemala: IV Censo Nacional Agropecuario, 2003. Online data: http://www.ine.gob.gt/np/agropecuario/tomo%20IV.pdf
Honduras	1993	1	2,077,460	Secretaría de Planificación, Coordinación y Presupuesto, Honduras: IV Censo Nacional Agropecuario 1993 (GLIMS data warehouse)
Jamaica	2009	1	113,338	Agricultural Business Information System, Jamaica: Livestock Summary Report, 2009 (GLIMS data warehouse)
Martinique	2000	1	28,342	Agreste la Statistique Agricole, Le Ministère de l'Agriculture, de l'Alimentation, de la Pêche et de la Ruralité, France: Recensement Agricole 2000. Online data: http://agreste.agriculture.gouv.fr/recensement-agricole-2010/resultats-donnees-chiffrees/
Mexico	2007	2	23,237,877	Instituto Nacional de Estadística Geografía e Informática, México: Censo Agrícola, Ganadero y Forestal 2007. Online data: http://www.inegi.org.mx/est/contenidos/proyectos/Agro/ca2007/Resultados_Agricola/default.aspx
Nicaragua	2001	2	2,657,039	Instituto Nacional de Estadística y Censos, Nicaragua: III Censo Nacional Agropecuario, 2001 (GLIMS data warehouse)

Panama	2001	2	1,531,914	Instituto Nacional de Estadística y Censo: Existencia de Animales en la República, por clase de animal, según provincia: 21 de abril de 1991 al 22 abril de 2001. Online data: http://www.contraloria.gob.pa/inec/Avance/Avance.aspx?ID_CATEGORIA=2&ID_CIFRAS=8&ID_IDIOMA=1
Puerto Rico	2007	2	259,918	National Agricultural Statistics Service - United States Department of Agriculture: National Agricultural Census 2007. Online data: http://www.agcensus.usda.gov/Publications/2007/Full_Report/
Saint Lucia	2007	6	5,345	Ministry of Agriculture, Forestry and Fisheries: Census of Agriculture - Final report 2007. Online data: http://www.govt.lc/www/publications/AgricultureCensus2007.pdf
Saint Vincent and the Grenadines	2000	1	4,767	Ministry of Agriculture and Labour, Government of Saint Vincent and the Grenadines: National Agricultural Census 2000 (GLIMS data warehouse)
United States of America	2007	2	96,184,610	National Agricultural Statistics Service - United States Department of Agriculture: National Agricultural Census 2007. Online data: http://www.agcensus.usda.gov/Publications/2007/Full_Report/

No Data for: Anguilla, Antigua and Barbuda, Bahamas, Barbados, Bermuda, Bird Island, British Virgin Islands, Dominica, Grenada, Haiti, Montserrat, Netherlands Antilles, Saint Kitts and Nevis, Turks and Caicos Islands, United States Virgin Islands

South America Tile

COUNTRY	YEAR	LEVEL	Totals in GLIMS	SOURCE
Argentina	2009	2	53,637,537	Instituto Nacional de Tecnología Agropecuaria, Argentina: Análisis de la actividad ganadera bovina de carne por estratos de productores y composición del stock. Años 2008 y 2009 (GLIMS data warehouse)
Bolivia, Plurinational State of	2008	1	8,189,599	Instituto Nacional de Estadística, Bolivia: Encuesta Nacional Agropecuaria. Online data: http://www.gobernacionlapaz.gob.bo/archivos/Sec_Deptal/SDPD/DID/Estadistica_Deptal/Agropecuario/Resultados_ENA_2008/EncuestaNacionalAgropecuaria.pdf
Brazil	2009	2	205,225,804	Istituto Brasileiro de Geografia e Estatistica: Producao da Pecuria Municipal 2009. Online data: http://www.ibge.gov.br/home/estatistica/economia/ppm/2009/default.shtm
Chile	2007	3	3,718,532	Instituto Nacional de Estadísticas, Chile: Censo Agropecuario y Forestal 2007. Online data: http://www.ine.cl/canales/chile_estadistico/censos_agropecuarios/censo_agropecuario_07_comunas.php
Colombia	2009	1	22,787,372	Instituto Agropecuario, Colombia: Censo Agropecuario Nacional 2004 - 2009 (GLIMS data warehouse)
Ecuador	2000	2	4,481,052	Project SICA, Agricultural Information System, Ministry of Agriculture and Livestock, Ecuador: III Censo Nacional Agropecuario (GLIMS data warehouse)
Falkland Islands (Malvinas)	2010	1	4,738	Department of Agriculture, Falkland Islands Government: Statistics Book 2010 (GLIMS data warehouse)
French Guiana	2000	6	9,394	Agreste la Statistique Agricole, Le Ministère de l'Agriculture, de l'Alimentation, de la Pêche et de la Ruralité, France: Recensement Agricole 2000. Online data: http://agreste.agriculture.gouv.fr/recensement-agricole-2010/resultats-donnees-chiffrees/
Paraguay	2008	2	10,466,641	Ministerio de Agricultura y Ganadería, Paraguay: Censo Agropecuario Nacional 2008 (GLIMS data warehouse)
Peru	2010	1	5,520,197	Instituto Nacional de Estadística e Informática, Peru: Compendio Estadístico 2011 (GLIMS data warehouse)
Suriname	1994	1	123,630	Environmental Research Group Oxford, ERGO: ERGO, 1996 "Livestock Geography. A demonstration of GIS techniques applied to Global Livestock Systems and Populations", Animal Health Division, FAO, Rome (William Wint extracted from the agroecological zone estimates derived from the FAOSTAT data for 1994) (GLIMS data warehouse)
Uruguay	2010	1	11,092,285	Ministerio de Ganadería, Agricultura y Pesca, Estadísticas Agropecuarias, Uruguay: Datos de la Declaración Jurada de DICOSE 2010 - Datos Generales de Lechería y de Suinos (GLIMS data warehouse)
Venezuela, Bolivarian Republic	2007	2	13,302,976	Ministerio del Poder Popular para la Agricultura y Tierras - Venezuela: VII Censo Agrícola 2007. Online data: http://censo.mat.gob.ve/

No Data for: Aruba, Guyana, Trinidad and Tobago

Oceania Tile

COUNTRY	YEAR	LEVEL	Totals in GLIMS	SOURCE
American Samoa	2003	1	300	National Agricultural Statistics Service - United States Department of Agriculture: National Agricultural Census 2007. Online data: http://www.agcensus.usda.gov/Publications/2007/Full_Report/
Australia	2007	6	26,113,333	Australian Bureau of Statistics: Agricultural commodities: small area data, Australia 2006-2007 (GLIMS data warehouse)
New Zealand	2010	1	9,617,244	Statistics New Zealand: 2011 Livestock numbers by Regional Council. Online data: http://www.stats.govt.nz/tools_and_services/nzdotstat/agriculture-statistics.aspx
Northern Mariana Islands	2007	1	1,395	National Agricultural Statistics Service - United States Department of Agriculture: National Agricultural Census 2007. Online data: http://www.agcensus.usda.gov/Publications/2007/Full_Report/
Papua New Guinea	1989	1	62,888	National Statistical Office: Agricultural Large Holdings 1989 (GLIMS data warehouse)
Tonga	2001	1	10,354	Ministry of Agriculture and Forestry, Kingdom of Tonga: Agriculture Census 2001. Online data: http://www.spc.int/prism/tonga/index.php/surveys/agriculture-census-2011

No Data for: Ashmore and Cartier Islands, Cook Islands, Fiji, French Polynesia, Guam, Kiribati, Marshall Islands, Federated States of Micronesia, Nauru, New Caledonia, Niue, Norfolk Island, Palau, Pitcairn, Samoa, Solomon Islands, Tokelau, Tuvalu, Vanuatu, Wake Island, Wallis and Futuna

Supplementary information 5b – Country details sub-national statistics for pigs (year and level of observation) by continental tile.

Africa Tile

COUNTRY	YEAR	LEVEL	Totals in GLIMS	SOURCE
Algeria	2002	1	5,702	Direction des Services Vétérinaires - Ministère de l'Agriculture et du Développement Rural 2003 (GLIMS data warehouse)
Angola	2009	1	3,984,614	CountrySTAT - Food and agriculture data network: Número de animais vivos by Ano, Especie, Nivel administrativo. Online data: http://countrystat.org/home.aspx?c=AGO&ta=007SPD035&tr=21
Benin	2008	2	329,700	CountrySTAT - Food and agriculture data network: Élevage - Total des effectifs d'animaux vivants 2010-2012. Online data: http://www.countrystat.org/home.aspx?c=BEN&ta=053SPD135&tr=21
Botswana	2009	2	3,302	Central Statistics Office of Botswana: 2009 and 2010 Annual Agricultural Surveys (GLIMS data warehouse)
Burkina Faso	2008	2	2,083,127	Ministère de l'Economie et de Développement, Direction General des Prévisions et des Statistiques Agricoles: Les régions en chiffres, 2008 & 2010 (GLIMS data warehouse)
Burundi	2005	2	156,138	Direction Provinciale de l'Agriculture et de l'Élevage, Burundi: Monographies des les Communes 2006 (GLIMS data warehouse))
Cameroon	2007	2	218,080	CountrySTAT - Food and agriculture data network (GLIMS data warehouse)
Cape Verde	2004	2	77,316	Directorate of livestock, Cape Verde: Recenseamento Geral da Agricultura, 2004 (GLIMS data warehouse)
Central African Republic	1990	1	441,480	Environmental Research Group Oxford, ERGO: FAO, 2004. Agro-ecological distributions for Africa (GLIMS data warehouse)
Chad	2007	1	39,220	Direction de l'Élevage et des Ressources Animales: Effectifs du cheptel national par région administrative 2007 (GLIMS data warehouse)
Congo	2000	1	15,455	Ministère de l'Agriculture et de l'Élevage: Enquête Agricole Pilote, Campagne Agricole 1999-2000 (GLIMS data warehouse)
Côte d'Ivoire	2001	1	476,708	Ministère de l'Agriculture et des Ressources Animales, Direction de la Programmation, Côte d'Ivoire: Recensement National de l'Agriculture 2001 (GLIMS data warehouse)
Democratic Republic of the Congo	2009	1	960,305	Centre for Ticks and Tick-Borne Diseases: Preliminary census data from SADC and Eastern Africa (GLIMS data warehouse)
Eritrea	2002	2	2,203	Environmental Research Group Oxford, ERGO: GIEWS special reports. Online data: http://www.fao.org/docrep/005/y7678e/y7678e00.htm
Ethiopia	2002	3	28,598	Central Statistical Authority of Ethiopia: Agricultural Census 2001-2002 (GLIMS data warehouse)
Gabon	2002	1	213,000	Environmental Research Group Oxford, ERGO: FAO, 2004. Agro-ecological distributions for Africa (GLIMS data warehouse)

Gambia	2007	1	24,281	National Agriculture Development Agency, Gambia: National Agricultural Sample Survey Report (NASS), Oct. 2008 (GLIMS data warehouse)
Ghana	1996	1	354,678	Veterinary Services of Ghana: Livestock Census, 1994-1996 (GLIMS data warehouse)
Guinea	2002	2	97,205	Service National des Statistiques Agricoles, République de Guinée 2002 (GLIMS data warehouse)
Guinea-Bissau	1994	2	204,375	Instituto Nacional de Estadistica: Inquerito Annual sobre Superficie Rendimento e Producao. Campanha 93/94 (GLIMS data warehouse)
Kenya	2005	3	310,154	Ministry of Livestock Development, Kenya: Compilation of divisional data 2005 (GLIMS data warehouse)
Lesotho	2008	1	83,976	Bureau of Statistics of Lesotho: Livestock winter 2008/09 Tables. Online data: http://www.bos.gov.ls/Downloads.htm
Madagascar	2002	3	460,805	Direction des Ressources Animales: Situation de l'élevage a Madagascar 2002 (GLIMS data warehouse)
Malawi	2007	2	792,364	Agriculture Statistics Division of the National Statistical Office of Malawi (NSO): National Census of Agriculture and Livestock (October 2006 - October 2007) (GLIMS data warehouse)
Mali	2010	1	75,089	CountrySTAT - Food and agriculture data network: Élevage - Total des effectifs d'animaux vivants. Online data: http://www.countrystat.org/home.aspx?c=MLI&tr=21
Mauritius	2010	1	22,327	Central Statistic Office of Mauritius: Digest of Agricultural Statistics (July 2010) (GLIMS data warehouse)
Morocco	2002	2	4,783	Ministère de l'Agriculture, du Développement Rural et des Pêches Maritimes: Evolution des effective au niveau des provinces (GLIMS data warehouse)
Mozambique	2010	1	1,340,712	National Directorate of Livestock: Censo Agropecuario 2009-2010 (GLIMS data warehouse)
Namibia	2006	1	52,485	FAO Special Report, FAO/WFP Crop, Livestock and Food Security Assessment Mission to Namibia (GLIMS data warehouse)
Niger	2002	1	39,000	Ministère des Ressources Animales: Revue des statistiques courantes sur l'élevage au Niger (GLIMS data warehouse)
Nigeria	2007	1	2,444,096	National Bureau of Statistics, Nigeria: Agricultural Census 2007 (GLIMS data warehouse)
Réunion	2000	2	70,921	Agreste la Statistique Agricole, Le Ministère de l'Agriculture, de l'Alimentation, de la Pêche et de la Ruralité, France: Recensement Agricole 2000. Online data: http://agriculture.gouv.fr/recensement-agricole-2010
Rwanda	2008	2	310,834	National Institute of Statistics of Rwanda: National Agricultural Survey 2008 (Final report) (GLIMS data warehouse)
Saint Helena	2009	1	773	Agriculture and Natural Resources Department of St. Helena: Livestock Census 2009 (GLIMS data warehouse)
Sao Tome and Principe	1997	2	13,495	Ministère de l'Agriculture et de la Pèche: Enquête Agricole de Production 1997 (GLIMS data warehouse)
Senegal	2009	2	344,172	Agence Nationale de la Statistique et de la Démographie du Sénégal: Live Data Base (LDB). Online data: http://www.ansd.sn/publications_SES_region.html
Sierra Leone	2004	2	65,423	Ministry of Agriculture & Food Security of Sierra Leone: 2004 Populations and Housing Census Statistical Office (GLIMS data warehouse)

Somalia	2002	1	4,002	Environmental Research Group Oxford, ERGO: FAO, 2004. Agro-ecological distributions for Africa (GLIMS data warehouse)
South Africa	2002	2	1,979,931	Directorate Veterinary Services, South Africa: Animal Disease Report, January to September 2003 (GLIMS data warehouse)
Swaziland	2010	1	38,915	Directorate of Veterinary and Livestock Services: Swaziland Livestock Statistics, Census 2010 (GLIMS data warehouse)
Togo	2002	1	243,241	Data from Dr. Guy Hendrickx (GLIMS data warehouse)
Uganda	2000	4	770,319	Uganda Bureau of Statistics: Uganda Census 2000 (GLIMS data warehouse)
United Republic of Tanzania	2008	2	1,592,600	CountrySTAT - Food and agriculture data network (GLIMS data warehouse)
Zambia	2009	1	517,553	Department of Research and Specialist Services, Zambia: National Livestock Census by Province 1994 to 2009 (GLIMS data warehouse)
Zimbabwe	2008	2	202,234	Department of Livestock and Veterinary Services: Livestock Census Summary (GLIMS data warehouse)

No Data for: Bassas da India, British Indian Ocean Territory, Comoros, Djibouti, Egypt, Equatorial Guinea, Europa Island, Glorioso Island, Juan de Nova Island, Liberia, Libya, Mauritania, Mayotte, Seychelles, Sudan, Tromelin Island, Tunisia, Western Sahara

Asia Tile

COUNTRY	YEAR	LEVEL	Totals in GLIMS	SOURCE
Armenia	2011	1	114,777	National statistical service of the republic of Armenia: Atlas the Republic of Armenia by Regions and the Yerevan City, 2011. Online data: http://www.armstat.am/en/?nid=50
Azerbaijan	2011	1	6,146	The State Statistical Committee of the Republic of Azerbaijan: The data on regions. Online data: http://www.stat.gov.az/source/agriculture/indexen.php
Bangladesh	2009	2	206,872	FAO - Emergency Center for Transboundary Animal Diseases (GLIMS data warehouse)
Belarus	2011	2	2,954,600	National Statistical Committee of the Republic of Belarus: Agriculture of the Republic of Belarus, 2011 (GLIMS data warehouse)
Bhutan	2009	2	21,532	Ministry of agriculture and forest, Royal government of Bhutan: Dzongkhag livestock sector (GLIMS data warehouse)
Bulgaria	2010	6	664,000	Ministry of Agriculture and Forestry - Agrostatistics Directorate, Bulgaria: Agricultural Census 2010. Online data: http://www.mzh.government.bg/MZH/ShortLinks/SelskaPolitika/Agrostatistics/Structure_agricultural_holdings/Results.aspx
Cambodia	2011	2	2,076,990	Ministry of Agriculture, Forestry and Fisheries: Cambodia Livestock Census 2011 (GLIMS data warehouse)
China, mainland	2006	3	510,417,525	National Bureau of Statistics of China: China Statistical Yearbook, 2006. For Taiwan: Statistics Office, COA, Executive Yuan, Taiwan Statistical Yearbook, 2010 (GLIMS data warehouse)
Cyprus	2003	1	443,040	Statistical Service of Cyprus: Census of Agriculture 2003. Online data: http://www.mof.gov.cy/mof/cystat/statistics.nsf/All/DE20AEA98C4DD6EEC22577A50041F0B8/$file/Census_of_Agriculture-2003.pdf?OpenElement
Georgia	2005	2	480,695	AgroWeb Central Asia and Caucasus: Georgian National Association for Animal Production (GNAAP), 2005 (GLIMS data warehouse)
India	2007	2	10,501,506	Department of Animal Husbandry, Dairying & Fisheries, Ministry of Agriculture, India: Census of Agriculture, 2007 (GLIMS data warehouse)
Indonesia	2011	1	5,854,781	Department Pertanian Republik Indonesia: Buku Statistik, 2012 (GLIMS data warehouse)
Japan	2009	1	9,902,400	Statistics Department, Minister's Secretariat, Ministry of Agriculture, Forestry and Fisheries: Japan Statistical Yearbook 2010. Online data: http://www.maff.go.jp/e/tokei/kikaku/nenji_e/85nenji/index.html
Kazakhstan	2004	1	1,341,300	Agency on Statistics of the Republic of Kazakhstan: Main socio economic indicators of regions in Kazakhstan 2005. Almaty, Kazakhstan (GLIMS data warehouse)
Kyrgyzstan	2003	1	105,476	National Statistic Committee of Kyrgyzstan: Results of the first agricultural census of the Kyrgyz Republic of 2003 (Second Stage) (GLIMS data warehouse)

Lao People's Democratic Republic	2009	2	2,663,363	Department of Livestock and Fisheries, Laos: 2009 district data (David Bourn, " Environmental Animal Health Management Initiative for Enhanced Smallholder Production" , 2010) (GLIMS data warehouse)
Malaysia	2001	1	2,038,278	Department of Veterinary Service, Malaysia: Livestock Products statistics 1996-2002 (GLIMS data warehouse)
Myanmar	2011	3	10,248,983	Livestock Breeding and Veterinary Department (LBVD), Ministry of Livestock Breeding and Fisheries, Yangon, Myanmar: Myanmar Farm Animal Resources: Numbers and Distributions from Administrative Returns, 2011/12 (GLIMS data warehouse)
Nepal	2009	3	1,021,122	Ministry of Agriculture and Cooperatives (MOAC), Nepal: 2009 data (GLIMS data warehouse)
Philippines	2010	3	12,793,664	Bureau of Agricultural Statistics (BAS): Livestock Survey Philippines 2010 (GLIMS data warehouse)
Republic of Korea	2005	1	8,961,505	Ministry of Agriculture & Forestry. Republic of Korea: Agricultural and Forestry Statistical Yearbook 2005 (GLIMS data warehouse)
Republic of Moldova	2010	2	377,100	Statistica Moldovei: Livestock in all categories of producers in territorial aspect, 2010. Online data: http://www.statistica.md/pageview.php?l=en&idc=349
Romania	2011	1	5,363,797	National Institute of Statistics, Romania: Tempo online database/Agriculture/Livestock and Animal Production 2000-2011. Online data: https://statistici.insse.ro/shop/?lang=en
Russian Federation	2011	1	18,634,550	Russian Federation - Federal State Statistical Service: Livestock and poultry production, 2011 and 2012. Online data: http://www.fedstat.ru/indicators/start.do
Sri Lanka	2011	2	82,030	Agriculture and Environment Statistics Division, Department of Census and Statistics, Sri Lanka: Livestock Population by Type and by District - 2003 – 2012. Online data: http://www.statistics.gov.lk/agriculture/Livestock/LivestockPopulationSubnational.html
Tajikistan	2009	2	419	Agency for Statistics, Tajikistan Republic: Regions of Tajikistan Republic, 2010 (GLIMS data warehouse)
Thailand	2006	1	11,763,705	Regional Data Exchange System on Food and Agricultural Statistics in Asia and Pacific Countries: Thailand sub national Statistics. Online data: http://www.faorap-apcas.org/thailand.html
Turkey	2009	2	1,896	Turkish Statistical Institute: Livestock statistics database 2009 (GLIMS data warehouse)
Ukraine	2011	1	7,960,400	State statistics committee Ukraine: Head of livestock and poultry by regions (GLIMS data warehouse)
Uzbekistan	2001	1	81,600	State Statistics Department of Uzbekistan: Agriculture of Uzbekistan, 2001. Minmakroeconomstat of Republic Uzbekistan, State Statistics Department of Uzbekistan Tashkent, 2002 (GLIMS data warehouse)
Viet Nam	2001	6	21,445,693	General Statistics Office of Vietnam: Agricultural Census database 2001. Online data: http://www.gso.gov.vn/default_en.aspx?tabid=477&idmid=4&ItemID=1824

No Data for: Afghanistan, Bahrain, Brunei Darussalam, Christmas Island, Cocos (Keeling) Islands, Democratic People's Republic of Korea, Islamic Republic of Iran, Iraq, Israel, Jordan, Kuril Islands, Kuwait, Lebanon, Liancourt Rock, Maldives, Mongolia, Oman, Pakistan, Paracel Islands, Qatar, Saudi Arabia, Scarborough Reef, Senkaku Islands, Singapore, Spratly Islands, Syrian Arab Republic, Timor-Leste, Turkmenistan, United Arab Emirates, Yemen

Europe Tile

COUNTRY	YEAR	LEVEL	Totals in GLIMS	SOURCE
Albania	2010	1	164,500	Institute of Statics, Albania: Agricultural sector, Livestock 2001-2010 (GLIMS data warehouse)
Austria	2010	1	3,134,156	Statistics Austria: Livestock Survey, 2010 (GLIMS data warehouse)
Belgium	2010	2	6,429,600	EUROSTAT online database. General Statistics, Region, Agriculture, Animal Populations and Land Use. Online data: http://epp.eurostat.ec.europa.eu/portal/page/portal/agriculture/data/
Bosnia and Herzegovina	2009	6	529,095	Federal Office of Statistics of Federation of Bosnia and Herzegovina: Number of animals from Federal Agro-Mediterranean Institute of Mostar (GLIMS data warehouse)
Croatia	2003	1	1,726,895	Central bureau of statistics, Republic of Croatia: Agricultural Census 2003. Online data: http://www.dzs.hr/default_e.htm
Czech Republic	2010	2	1,906,929	Czech Statistical Office: Agrocensus regions: Farm Structure Survey and Survey on Agricultural Production Methods 2010. Online data: http://www.czso.cz/csu/2012edicniplan.nsf/engp/2127-12
Denmark	2010	2	13,173,060	Denmark Statistics: Farms by region and selected farms, crops and livestock. Online data: http://www.statistikbanken.dk/statbank5a/SelectVarVal/Define.asp?Maintable=BDF51&PLanguage=1
Estonia	2001	2	327,195	Statistikaamet Statistical Office of Estonia: Agricultural Census 2001. Online data: http://www.stat.ee/agricultural-census-2001
Finland	2010	6	1,366,800	The Information Centre of the Ministry of Agriculture and Forestry, Finland: Yearbook of Farm Statistics 2011 (GLIMS data warehouse)
France	2009	2	13,245,288	Service de la Statistique et de la Prospective (SSP): Statistique Agricole Annuelle (GLIMS data warehouse)
Germany	2007	2	25,247,881	Federal Statistical Office, Germany: GENESIS database. Online data: https://www.regionalstatistik.de/genesis/online/online;jsessionid=EE45147898822814978BE734145275C4?operation=sprachwechsel&option=en
Greece	2007	2	1,111,540	Hellenic Statistic Authority (EL.STAT): Farms and number of animals by type, region and county (GLIMS data warehouse)
Hungary	2010	1	3,169,000	Hungarian Central Statistical Office: Regional Statistics. Online data: http://www.ksh.hu/agriculture
Iceland	2010	2	3,615	Statistics Iceland: 2010 data (GLIMS data warehouse)
Ireland	2000	1	1,722,108	Central Statistical Office Ireland: Census of Agriculture 2000. Online data: http://www.cso.ie/en/releasesandpublications/agricultureandfishing/censusofagriculturemainresultsjune2000/
Italy	2010	3	9,331,143	Istituto Nazionale di Statistica: Censimento Agricoltura 2010. Online data: http://dati-censimentoagricoltura.istat.it
Latvia	2001	1	368,909	Central Statistical Bureau of Latvia: Results of 2001 Agricultural Census. Online data: http://www.csb.gov.lv/en/dati/agricultural-census-2001-30765.html

Liechtenstein	2009	6	1,811	Amt für Statistik Liechtenstein: Landwirtschaftsstatistik 2009 (GLIMS data warehouse)
Lithuania	2010	2	942,163	Lietuvos Statistikas Departamenta: Number of livestock by administrative territory, 2011 (GLIMS data warehouse)
Luxembourg	2008	2	77,841	Portail des statistiques du Grand-Duché de Luxembourg (STATEC): Recensement Agricole, 2008 (GLIMS data warehouse)
Malta	2010	6	70,593	National Statistics Office of Malta: Census of Agriculture 2010. Online data: http://www.nso.gov.mt/statdoc/document_view.aspx?id=3287&backurl=/themes/theme_page.aspx
Montenegro	2010	1	41,118	Statistical office of Montenegro (MONSTAT): Agriculture Census 2010. Online data: http://www.monstat.org/eng/page.php?id=58&pageid=58
Netherlands	2010	2	12,254,972	Centraal Bureau voor de Statistiek, Netherlands: Agriculture: crops, livestock and land use by general far type, region - 2011 (GLIMS data warehouse)
Norway	2010	1	1,607,772	Statistics Norway: Agriculture Statistics 2011 (GLIMS data warehouse)
Poland	2009	1	14,278,600	Central Statistical Office, Poland: Statistical Yearbook of Agriculture 2010 (GLIMS data warehouse)
Portugal	2009	2	1,851,290	Instituto Nacional de Estatística - Statistics Portugal: Agricultural Census 2009. Online data: http://ra09.ine.pt/xportal/xmain?xpid=RA2009&xpgid=ra_home
Serbia	2006	1	3,998,927	Statistical Office of Serbia: Municipalities of Serbia 2007 (GLIMS data warehouse)
Slovakia	2009	2	740,862	Statistical Office of the Slovak Republic: Livestock by territory, products and period. Online data: http://px-web.statistics.sk/PXWebSlovak/DATABASE/En/databasetree.asp
Slovenia	2010	2	308,540	Statistical Office of the Republic of Slovenia: Agricultural Census Slovenia 2010. Online data: http://www.stat.si/eng/novica_prikazi.aspx?id=4594
Spain	2010	2	25,704,041	Ministerio de Medio Ambiente y Medio Rural y Marino, Gobierno de España (MARM): Encuestas Ganaderas, 2010 (GLIMS data warehouse)
Sweden	2009	1	1,528,740	Statistics Sweden: Yearbook of Agricultural Statistics 2010 (GLIMS data warehouse)
Switzerland	2009	1	1,557,204	Swiss Federal Statistical Office: Recensement des entreprises agricoles,1999-2009 (GLIMS data warehouse)
The former Yugoslav Republic of Macedonia	2006	2	173,185	State Statistic Office of the Republic of Macedonia: Census of Agriculture 2007. Online data: http://www.stat.gov.mk/PrikaziPublikacija_en.aspx?id=51&rbr=205
United Kingdom	2009	2	4,525,710	Office for National Statistics, U.K.: Statistics by Region. Online data: http://www.statistics.gov.uk/hub/regional-statistics/

No Data for: Andorra, Faroe Islands, Gibraltar, Guernsey, Holy See, Isle of Man, Jersey, Madeira Islands, Monaco, San Marino, Svalbard and Jan Mayen Islands

North America Tile

COUNTRY	YEAR	LEVEL	Totals in GLIMS	SOURCE
Belize	2003	1	21,224	Ministry Of Agriculture, Fisheries and Cooperatives, Belize: Annual Report 2003. Online data: http://www.agriculture.gov.bz/PDF/Annual_Report_2003.pdf
Canada	2006	2	14,168,313	Statistics Canada: 2006 Census of Agriculture. Online data: http://www.statcan.gc.ca/ca-ra2006/index-eng.htm
Cayman Islands	2009	1	1,063	Economics and Statistics Office - Government of the Cayman Islands: Statistical Compendium 2009 (GLIMS data warehouse)
Costa Rica	2006	1	297,359	Ministerio de Agricultura y Ganadería, Costa Rica: Programa Nacional de Sanidad Porcina 2006 (GLIMS data warehouse)
El Salvador	2007	2	165,866	Ministerio de Economía - El Salvador: IV Censo Agropecuario 2007-2008 (GLIMS data warehouse)
Guadeloupe	2000	1	25,839	Agreste la Statistique Agricole, Le Ministère de l'Agriculture, de l'Alimentation, de la Pêche et de la Ruralité, France: Recensement Agricole 2000. Online data: http://agreste.agriculture.gouv.fr/recensement-agricole-2010/resultats-donnees-chiffrees/
Guatemala	2003	2	419,170	Instituto Nacional de Estadística, Guatemala: IV Censo Nacional Agropecuario 2003. Online data: http://www.ine.gob.gt/np/agropecuario/tomo%20IV.pdf
Jamaica	2009	1	361,540	Agricultural Business Information System, Jamaica: Livestock Summary Report, 2009 (GLIMS data warehouse)
Martinique	2000	1	20,621	Agreste la Statistique Agricole, Le Ministère de l'Agriculture, de l'Alimentation, de la Pêche et de la Ruralité, France: Recensement Agricole 2000. Online data: http://agreste.agriculture.gouv.fr/recensement-agricole-2010/resultats-donnees-chiffrees/
Mexico	2007	2	8,964,101	Instituto Nacional de Estadística Geografía e Informática, México: Censo Agrícola, Ganadero y Forestal 2007. Online data: http://www.inegi.org.mx/est/contenidos/proyectos/Agro/ca2007/Resultados_Agricola/default.aspx
Nicaragua	2001	2	383,172	Instituto Nacional de Estadística y Censos, Nicaragua: III Censo Nacional Agropecuario 2001 (GLIMS data warehouse)
Panama	2001	2	312,102	Instituto Nacional de Estadística y Censo: Existencia de animales en la República, por clase de animal, según provincia: 21 de abril de 1991 al 22 abril de 2001. Online data: http://www.contraloria.gob.pa/inec/Avance/Avance.aspx?ID_CATEGORIA=2&ID_CIFRAS=8&ID_IDIOMA=1
Puerto Rico	2007	2	63,585	National Agricultural Statistics Service - United States Department of Agriculture: National Agricultural Census 2007. Online data: http://www.agcensus.usda.gov/Publications/2007/Full_Report/
Saint Lucia	2007	6	19,520	Ministry of Agriculture, Forestry and Fisheries: Census of Agriculture - Final report 2007. Online data: http://www.govt.lc/www/publications/AgricultureCensus2007.pdf

Saint Vincent and the Grenadines	2000	1	4,807	Ministry of Agriculture and Labour, Government of Saint Vincent and the Grenadines: National Agricultural Census 2000 (GLIMS data warehouse)
United States of America	2007	2	67,239,043	National Agricultural Statistics Service - United States Department of Agriculture: National Agricultural Census 2007. Online data: http://www.agcensus.usda.gov/Publications/2007/Full_Report/

No Data for: Anguilla, Antigua and Barbuda, Bahamas, Barbados, Bermuda, Bird Island, British Virgin Islands, Cuba, Dominica, Dominican Republic, Grenada, Haiti, Honduras, Montserrat, Netherlands Antilles, Saint Kitts and Nevis, Turks and Caicos Islands, United States Virgin Islands

South America Tile

COUNTRY	YEAR	LEVEL	Totals in GLIMS	SOURCE
Argentina	2002	2	2,184,445	Instituto Nacional de Estadística y Censos. Argentina: Censo Nacional Agropecuario 2002 (CNA 2002). Online data: http://www.indec.gov.ar/agropecuario/cna_principal.asp
Bolivia, Plurinational State of	2008	1	2,640,615	Instituto Nacional de Estadística, Bolivia: Encuesta Nacional Agropecuaria. Online data: http://www.gobernacionlapaz.gob.bo/archivos/Sec_Deptal/SDPD/DID/Estadistica_Deptal/Agropecuario/Resultados_ENA_2008/EncuestaNacionalAgropecuaria.pdf
Brazil	2009	2	38,045,454	Istituto Brasileiro de Geografia e Estatistica: Producao da Pecuria Municipal 2009. Online data: http://www.ibge.gov.br/home/estatistica/economia/ppm/2009/default.shtm
Chile	2007	3	2,928,606	Instituto Nacional de Estadísticas, Chile: Censo Agropecuario y Forestal 2007. Online data: http://www.ine.cl/canales/chile_estadistico/censos_agropecuarios/censo_agropecuario_07_comunas.php
Colombia	2009	1	3,908,962	Instituto Agropecuario, Colombia: Censo Agropecuario Nacional 2004 - 2009 (GLIMS data warehouse)
Ecuador	2009	6	1,042,022	Instituto Nacional de Estadística y Censos, Ecuador: Encuesta Nacional de Superficie y Producción Agropecuaria Continua (ESPAC) (GLIMS data warehouse)
Falkland Islands (Malvinas)	2010	1	89	Department of Agriculture, Falkland Islands Government: Statistics Book 2010 (GLIMS data warehouse)
French Guiana	2000	6	8,087	Agreste la Statistique Agricole, Le Ministère de l'Agriculture, de l'Alimentation, de la Pêche et de la Ruralité, France: Recensement Agricole 2000. Online data: http://agreste.agriculture.gouv.fr/recensement-agricole-2010/resultats-donnees-chiffrees/
Paraguay	2008	2	1,072,655	Ministerio de Agricultura y Ganadería, Paraguay: Censo Agropecuario Nacional 2008 (GLIMS data warehouse)
Peru	2006	1	3,034,134	Instituto Nacional de Estadística e Informática, Peru: Compendio Estadístico 2007 (GLIMS data warehouse)
Suriname	1994	1	42,639	Environmental Research Group Oxford, ERGO: ERGO, 1996 "Livestock Geography. A demonstration of GIS techniques applied to Global Livestock Systems and Populations", Animal Health Division, FAO, Rome (William Wint extracted from the agroecological zone estimates derived from the FAOSTAT data for 1994) (GLIMS data warehouse)
Trinidad and Tobago	2010	6	35,273	Central Statistical Office - Ministry of Planning & Development Government of the Republic of Trinidad & Tobago: Pig Bulletin 2010 (GLIMS data warehouse)
Uruguay	2010	1	177,914	Ministerio de Ganadería, Agricultura y Pesca, Estadísticas Agropecuarias, Uruguay: Datos de la Declaración Jurada de DICOSE 2010 - Datos Generales de Lechería y de Suinos (GLIMS data warehouse)
Venezuela, Bolivarian Republic	2007	2	2,787,338	Ministerio del Poder Popular para la Agricultura y Tierras - Venezuela: VII Censo Agrícola 2007. Online data: http://censo.mat.gob.ve/

No Data for: Aruba, Guyana

Oceania Tile

COUNTRY	YEAR	LEVEL	Totals in GLIMS	SOURCE
American Samoa	2008	1	16,904	National Agricultural Statistics Service - United States Department of Agriculture: National Agricultural Census 2007. Online data: http://www.agcensus.usda.gov/Publications/2007/Full_Report/
Australia	2007	6	2,469,334	Australian Bureau of Statistics: Agricultural commodities: small area data, Australia 2006-2007 (GLIMS data warehouse)
New Caledonia	2002	2	25,447	Institut de la Statistique et des Etudes Economiques (ISEE): Recensement Général Agricole (RGA) 2002. Online data: http://www.davar.gouv.nc/portal/page/portal/davar/secteur_rural/statistiques_agricoles/donnees_synthese/recensement_agricole
New Zealand	2010	1	302,073	Statistics New Zealand: 2011 livestock numbers by Regional council. Online data: http://www.stats.govt.nz/tools_and_services/nzdotstat/agriculture-statistics.aspx
Northern Mariana Islands	2007	1	2,242	National Agricultural Statistics Service - United States Department of Agriculture: National Agricultural Census 2007. Online data: http://www.agcensus.usda.gov/Publications/2007/Full_Report/
Tonga	2001	1	113,580	Ministry of Agriculture and Forestry, Kingdom of Tonga: Agriculture Census 2001. Online data: http://www.spc.int/prism/tonga/index.php/surveys/agriculture-census-2011

No Data for: Ashmore and Cartier Islands, Cook Islands, Fiji, French Polynesia, Guam, Kiribati, Marshall Islands, Federated States of Micronesia, Nauru, Niue, Norfolk Island, Palau, Papua New Guinea, Pitcairn, Samoa, Solomon Islands, Tokelau, Tuvalu, Vanuatu, Wake Island, Wallis and Futuna

Supplementary information 5c – Country details on sub-national statistics for chickens (year and level of observation) by continental tile.

Africa Tile

COUNTRY	YEAR	LEVEL	Totals in GLIMS	SOURCE
Angola	2010	1	17,118,618	CountrySTAT - Food and agriculture data network: Número de animais vivos by Ano, Especie, Nivel administrativo 1. Online data: http://countrystat.org/home.aspx?c=AGO&ta=007SPD035&tr=21
Benin	2009	2	15,286,300	CountrySTAT - Food and agriculture data network: Élevage - Total des effectifs d'animaux vivants 2010-2012. Online data: http://www.countrystat.org/home.aspx?c=BEN&ta=053SPD135&tr=21
Botswana	2006	1	1,639,141	Central Statistics Office Republic of Botswana: Botswana Agricultural Census Report. Ministry of Finance and Development Planning, 2007 (GLIMS data warehouse)
Burkina Faso	2008	2	28,267,052	Ministère des Ressources Animales: Les Statistiques du Secteur de l'Élevage au Burkina Faso, 2003 & 2008 (GLIMS data warehouse)
Burundi	2007	1	1,267,675	CountrySTAT - Food and agriculture data network: Élevage - Total des effectifs d'animaux vivants. . Online data: http://www.countrystat.org/home.aspx?c=BDI&ta=029SPD035&tr=21
Côte d'Ivoire	2001	1	17,125,712	Ministère de l'Agriculture et des Ressources Animales, Direction de la Programmation, Côte d'Ivoire: Recensement National de l'Agriculture 2001 (GLIMS data warehouse)
Cameroon	2007	2	11,411,072	CountrySTAT - Food and agriculture data network (GLIMS data warehouse)
Cape Verde	2004	2	327,562	Directorate of Livestock, Cape Verde: Recenseamento Geral da Agricultura, 2004 (GLIMS data warehouse)
Central African Republic	1990	1	2,773,110	Environmental Research Group Oxford, ERGO: FAO, 2004. Agro-ecological distributions for Africa (GLIMS data warehouse)
Chad	2009	1	47,867,370	Ministère de l'Élevage et des Ressources Animales: Revue du secteur avicole (GLIMS data warehouse)
Comoros	2000	1	221,550	FAO Animal Production and Health Division: Rapport National Sur l'Etat des Ressources Génétiques Animales (GLIMS data warehouse)
Democratic Republic of the Congo	1994	1	20,561,809	Services national des Statistiques Agricoles 1994 (GLIMS data warehouse)
Egypt	2005	1	68,452,380	Economic Affaire Sector, Ministry of Agriculture and Land Reclamation, Egypt: Poultry in the 21st century (GLIMS data warehouse)
Ethiopia	2010	2	49,286,935	Central Statistical Authority, Ethiopia: Agricultural Sample Survey 2008 and 2010 (GLIMS data warehouse)
Gabon	2002	1	3,100,001	Environmental Research Group Oxford, ERGO: FAO, 2004. Agro-ecological distributions for Africa (GLIMS data warehouse)

Gambia	2007	1	820,438	National Agriculture Development Agency, Gambia: National Agricultural Sample Survey Report (NASS), Oct. 2008 (GLIMS data warehouse)
Ghana	2009	1	33,140,820	CountrySTAT - Food and agriculture data network (GLIMS data warehouse)
Guinea	2002	2	13,213,984	Service National des Statistiques Agricoles, République de Guinée 2002 (GLIMS data warehouse)
Guinea-Bissau	1994	2	729,029	Instituto Nacional de Estadística: Inquérito Anual sobre Superficie Rendimento e Producao. Campanha 93/94 (GLIMS data warehouse)
Kenya	2009	2	32,217,042	Kenya National Bureau of Statistics: Kenyan Livestock Population 2009 (GLIMS data warehouse)
Lesotho	2008	1	715,576	Bureau of Statistics of Lesotho: Livestock winter 2008/09 Tables. Online data: http://www.bos.gov.ls/Downloads.htm
Madagascar	2005	3	24,213,520	Ministère de l'Agriculture, Secrétariat General, Direction de la Prévision et des Etudes Economiques, Madagascar: Recensement de l'agriculture (RA) - Campagne agricole 2004-2005 (GLIMS data warehouse)
Malawi	2007	2	7,557,745	Agriculture Statistics Division of the National Statistical Office of Malawi (NSO): National Census of Agriculture and Livestock (October 2006 - October 2007) (GLIMS data warehouse)
Mali	2010	1	36,750,000	CountrySTAT - Food and agriculture data network: Élevage - Total des effectifs d'animaux vivants. Online data: http://www.countrystat.org/home.aspx?c=MLI&tr=21
Mozambique	2002	2	22,636,314	Direccao Nacional de Pecuaria, Mozambique: Annual Reports, 2003 (GLIMS data warehouse)
Namibia	2001	1	458,010	Department of Veterinary Services, Namibia: FAO Special Report, FAO/WFP Crop, Livestock and Food Security Assessment Mission to Namibia (GLIMS data warehouse)
Niger	2007	1	6,674,216	Ministère des Ressources Animales: Recensement Général de l'Agriculture et du Cheptel (RGAC 2005/2007). Online data: http://harvestchoice.org/publications/niger-recensement-general-de-lagriculture-et-du-cheptel-rgac-20052007-volume-v-producti
Nigeria	2006	1	52,383,612	National Bureau of Statistics, Federal Ministry of Agriculture and Rural Development: Estimated Subsistence Poultry Population by State (2006 estimates) (GLIMS data warehouse)
Rwanda	2008	1	2,894,972	Ministère de l'Agriculture et de l'élevage: FAO, Revue du secteur agricole: RWANDA 2008 (GLIMS data warehouse)
Réunion	2000	1	1,277,621	Agreste la Statistique Agricole, Le Ministère de l'Agriculture, de l'Alimentation, de la Pêche et de la Ruralité, France: Recensement Agricole 2000. Online data: http://agriculture.gouv.fr/recensement-agricole-2010
Saint Helena	2009	1	4,421	Agriculture and Natural Resources Department of St. Helena: Livestock Census 2009 (GLIMS data warehouse)
Sao Tome and Principe	1997	2	37,608	Ministère de l'Agriculture et de la Pêche: Enquête Agricole de Production, EAP 1997 (GLIMS data warehouse)
Senegal	2009	2	22,301,806	Agence Nationale de la Statistique et de la Démographie du Sénégal: Live Data Base (LDB). Online data: http://www.ansd.sn/publications_SES_region.html

Sierra Leone	2004	2	3,227,252	Ministry of Agriculture & Food Security of Sierra Leone: 2004 Populations and Housing Census Statistical Office (GLIMS data warehouse)
Somalia	2002	1	3,299,999	Environmental Research Group Oxford, ERGO: Agro-ecological distributions for Africa (GLIMS data warehouse)
South Africa	2002	1	45,400,346	Directorate Veterinary Services, South Africa: Animal Disease Report, January to September 2003 (GLIMS data warehouse)
Sudan	2005	2	35,024,922	Ministry of Animal Resources: Estimates supplied by veterinary staff at Ministry of Animal Resources for 2005 (GLIMS data warehouse)
Swaziland	2010	1	1,936,344	Directorate of Veterinary and Livestock Services: Swaziland Livestock Statistics, Census 2010 (GLIMS data warehouse)
Uganda	2000	4	12,759,176	Uganda Bureau of Statistics: Uganda census 2000 - Data acquired from W. Wint (GLIMS data warehouse)
United Republic of Tanzania	2009	2	26,839,376	CountrySTAT - Food and agriculture data network (GLIMS data warehouse)
Zambia	2008	1	10,421,085	Department of Research and Specialist Services, Zambia: National Livestock Census by Province 1994 to 2009 (GLIMS data warehouse)

No Data for: Algeria, Bassas da India, British Indian Ocean Territory, Congo, Djibouti, Equatorial Guinea, Eritrea, Europa Island, Glorioso Island, Juan de Nova Island, Liberia, Libya, Mauritania, Mauritius, Mayotte, Morocco, Seychelles, Togo, Tromelin Island, Tunisia, Western Sahara, Zimbabwe

Asia Tile

COUNTRY	YEAR	LEVEL	Totals in GLIMS	SOURCE
Afghanistan	2003	2	12,136,388	Ministry of Agriculture, Irrigation and Livestock of Afghanistan: Afghanistan - National Livestock Census 2002-2003 - Final Report. Online data: http://reliefweb.int/sites/reliefweb.int/files/resources/58C374339FC582F149256DF30009D60F-fao-afg-4dec.pdf
Bangladesh	2005	6	197,456,543	Bangladesh Bureau of Statistics: Data Retrieved 12 November 2009 from http://www.bbs.gov.bd/ (GLIMS data warehouse)
Belarus	2011	1	36,410,987	National Statistical Committee of the Republic of Belarus: Agriculture of the Republic of Belarus, 2011 (GLIMS data warehouse)
Bhutan	2008	1	198,148	National Statistics Bureau, Bhutan: Annual district statistics 2010 (GLIMS data warehouse)
Cambodia	2009	2	15,103,537	Department of Animal Production and Health, Cambodia: 2009 district data (David Bourn, "Environmental Animal Health Management Initiative for Enhanced Smallholder Production", 2010) (GLIMS data warehouse)
China	2005	6	5,012,700,208	Editorial Department of China Animal Husbandry: China animal husbandry yearbook. Beijing, China: China Agricultural Press (GLIMS data warehouse)
Georgia	2004	2	7,960,916	National Statistics Office of Georgia: First National Agricultural Census 2004 in Georgia. Online data: http://geostat.ge/cms/site_images/_files/english/agriculture/census/section7.pdf
India	2007	2	606,978,885	Department of Animal Husbandry, Dairying & Fisheries, Ministry of Agriculture, India: Census of Agriculture, 2007 (GLIMS data warehouse)
Indonesia	2010	1	1,349,625,877	Department Pertanian Republik Indonesia: Buku Statistik, 2011 (GLIMS data warehouse)
Japan	2009	1	285,349,000	Statistics Department, Minister's Secretariat, Ministry of Agriculture, Forestry and Fisheries: Japan Statistical Yearbook 2010. Online data: http://www.maff.go.jp/e/tokei/kikaku/nenji_e/85nenji/index.html
Jordan	2007	1	172,052	Department of Statistics, Jordan: The Agricultural Census 2007 (GLIMS data warehouse)
Kazakhstan	2004	1	25,480,500	Agency on Statistics of the Republic of Kazakhstan: Main socio economic indicators of regions in Kazakhstan 2005. Almaty, Kazakhstan (GLIMS data warehouse)
Kuwait	2000	1	26,314,863	Central Statistical Office and Public Authority for Agriculture and Fisheries: Annual Statistical Abstract 2000 (GLIMS data warehouse)
Kyrgyzstan	2003	1	3,585,842	National Statistic Committee of Kyrgyzstan: Results of the first agricultural census of the Kyrgyz Republic of 2003 (Second Stage) (GLIMS data warehouse)

Lao People's Democratic Republic	2009	2	16,047,773	Department of Livestock and Fisheries, Laos: 2009 district data (David Bourn, " Environmental Animal Health Management Initiative for Enhanced Smallholder Production" , 2010) (GLIMS data warehouse)
Lebanon	1997	2	12,053,000	United Nations, Economic and Social Commission for Western Asia, 1999: National farm data handbook for Lebanon. New York (GLIMS data warehouse)
Malaysia	2001	1	119,980,226	Department of Veterinary Service, Malaysia: Livestock Products statistics 1996-2002 (GLIMS data warehouse)
Myanmar	2006	6	82,154,065	Livestock Breeding and Veterinary Department (LBVD), Ministry of Livestock Breeding and Fisheries, Yangon, Myanmar: Myanmar Animal Census 2006 (GLIMS data warehouse)
Nepal	2009	3	24,333,826	Ministry of Agriculture and Cooperatives (MOAC), Nepal: 2009 data (GLIMS data warehouse)
Pakistan	1996	2	59,253,879	Agricultural Census Organization, Ministry of Food & Agriculture, Pakistan: Livestock census 1996 - special report. Lahore, Pakistan (GLIMS data warehouse)
Philippines	2006	3	135,912,594	Bureau of Animal Industry, Philippines: 2006 data (David Bourn, " Environmental Animal Health Management Initiative for Enhanced Smallholder Production" , 2010) (GLIMS data warehouse)
Republic of Korea	2004	1	109,627,646	Korea National Statistical Office: Korea Statistical Yearbook 2005 (GLIMS data warehouse)
Romania	2002	6	69,583,725	EUROSTAT on line database. Section: General Statistics, Region, Agriculture, Animal Populations and Land Use. February 2005. Online data: http://epp.eurostat.ec.europa.eu/portal/page/portal/agriculture/data/
Russian Federation	2006	1	216,620,630	Russian Federation - Federal State Statistical Service: Results of All-Russia Agricultural Census 2006 (1-8 vol.) (GLIMS data warehouse)
Saudi Arabia	2006	1	531,875,811	FAO Animal Production and Health Division: Saudi Arabia Poultry Sector Country Review (GLIMS data warehouse)
Sri Lanka	2002	2	15,617,153	Agriculture and Environment Statistics Division, Department of Census and Statistics, Sri Lanka: Census of Agriculture 2002, All Sectors. Online data: http://www.statistics.gov.lk/agriculture/AGC2002/AGC2002.htm
Syrian Arab Republic	2005	1	28,502,000	FAO Animal Production and Health Division, Emergency Centre for Transboundary Animal Diseases, Socio Economics, Production and Biodiversity Unit, 2008. Poultry sector country review: Syria (GLIMS data warehouse)
Tajikistan	2000	1	1,061,500	Government Statistic Committee of the Republic of Tajikistan: Agricultural Yearbook of the Republic of Tajikistan (GLIMS data warehouse)
Thailand	2004	3	191,375,293	Department of Livestock Development, Ministry of Agriculture and Cooperatives, Thailand: Census 2004 (X- ray surveys searching for HPAI outbreaks) (GLIMS data warehouse)
Turkey	2009	2	230,105,873	Turkish Statistical Institute: Livestock statistics database 2009 (GLIMS data warehouse)
Ukraine	2009	1	159,218,996	State Statistics Committee Ukraine: Head of livestock and poultry by regions (GLIMS data warehouse)

Viet Nam	2001	6	148,078,344	General Statistics Office of Vietnam: Agricultural Census database 2001. Online data: http://www.gso.gov.vn/default_en.aspx?tabid=477&idmid=4&ItemID=1824

No Data for: Armenia, Azerbaijan, Bahrain, Brunei Darussalam, Bulgaria, Christmas Island, Cocos (Keeling) Islands, Cyprus, Democratic People's Republic of Korea, Islamic Republic of Iran, Iraq, Israel, Kuril Islands, Liancourt Rock, Maldives, Mongolia, Oman, Paracel Islands, Qatar, Republic of Moldova, Scarborough Reef, Senkaku Islands, Singapore, Spratly Islands, Timor-Leste, Turkmenistan, United Arab Emirates, Uzbekistan, Yemen

Europe Tile

COUNTRY	YEAR	LEVEL	Totals in GLIMS	SOURCE
Albania	2010	1	5,244,500	Institute of Statics, Albania: Agricultural sector, Livestock 2001-2010 (GLIMS data warehouse)
Austria	2003	1	12,571,600	EUROSTAT online database. General Statistics, Region, Agriculture, Animal Populations and Land Use. Online data: http://epp.eurostat.ec.europa.eu/portal/page/portal/agriculture/data/
Belgium	2003	2	32,032,000	EUROSTAT online database. General Statistics, Region, Agriculture, Animal Populations and Land Use. Online data: http://epp.eurostat.ec.europa.eu/portal/page/portal/agriculture/data/
Croatia	2003	1	10,477,514	Central Bureau of Statistics, Republic of Croatia: Agricultural Census 2003. Online data: http://www.dzs.hr/default_e.htm
Czech Republic	2010	2	16,843,696	Czech Statistical Office: Agrocensus regions: Farm Structure Survey and Survey on Agricultural Production Methods 2010. Online data: http://www.czso.cz/csu/2012edicniplan.nsf/engp/2127-12
Denmark	2010	2	18,083,961	Denmark Statistics: Farms by region and selected farms, crops and livestock. Online data: http://www.statistikbanken.dk/statbank5a/SelectVarVal/Define.asp?Maintable=BDF51&PLanguage=1
Estonia	2001	2	1,611,794	Statistikaamet Statistical Office of Estonia: Agricultural Census 2001. Online data: http://www.stat.ee/agricultural-census-2001
Finland	2010	6	7,828,600	The Information Centre of the Ministry of Agriculture and Forestry, Finland: Yearbook of Farm Statistics 2011 (GLIMS data warehouse)
France	2001	2	205,617,000	Agreste la Statistique Agricole, Le Ministère de l'Agriculture, de l'Alimentation, de la Pêche et de la Ruralité: Agreste la Statistique Agricole, 2002 (GLIMS data warehouse)
Germany	2001	1	109,993,100	Federal Statistical Office, Germany: Statistical Yearbook 2003 for the Federal Republic of Germany (GLIMS data warehouse)
Greece	2007	2	33,495,026	Hellenic Statistic Authority (EL.STAT): Farms and number of animals by type, region and county (GLIMS data warehouse)
Hungary	2010	1	31,849,000	Hungarian Central Statistical Office: Regional Statistics. Online data: http://www.ksh.hu/agriculture
Iceland	2010	2	323,427	Statistics Iceland: 2010 data (GLIMS data warehouse)
Ireland	2000	1	12,724,881	Central Statistical Office Ireland: Census of Agriculture 2000. Online data: http://www.cso.ie/en/releasesandpublications/agricultureandfishing/censusofagriculturemainresultsjune2000/
Italy	2010	3	139,031,114	Istituto Nazionale di Statistica: Censimento Agricoltura 2010. Online data: http://dati-censimentoagricoltura.istat.it
Latvia	2001	1	3,470,101	Central Statistical Bureau of Latvia: Results of 2001 Agricultural Census. Online data: http://www.csb.gov.lv/en/dati/agricultural-census-2001-30765.html
Liechtenstein	2009	6	12,000	Amt für Statistik Liechtenstein: Landwirtschaftsstatistik 2009 (GLIMS data warehouse)

Lithuania	2010	2	9,465,819	Lietuvos Statistikas Departamenta: Number of livestock by administrative territory, 2011 (GLIMS data warehouse)
Luxembourg	2008	2	100,787	Portail des statistiques du Grand-Duché de Luxembourg (STATEC): Recensement Agricole, 2008 (GLIMS data warehouse)
Malta	2010	6	1,146,810	National Statistics Office of Malta: Census of Agriculture 2010. Online data: http://www.nso.gov.mt/statdoc/document_view.aspx?id=3287&backurl=/themes/theme_page.aspx
Montenegro	2010	1	411,083	Statistical office of Montenegro (MONSTAT): Agriculture Census 2010. Online data: http://www.monstat.org/eng/page.php?id=58&pageid=58
Netherlands	2010	2	101,247,711	Centraal Bureau voor de Statistiek, Netherlands: Agriculture: crops, livestock and land use by general far type, region - 2011 (GLIMS data warehouse)
Norway	2002	1	3,137,661	Statistics Norway: Statistical Yearbook 2003 (GLIMS data warehouse)
Poland	2009	1	124,129,100	Central Statistical Office, Poland: Statistical Yearbook of Agriculture 2010 (GLIMS data warehouse)
Portugal	2009	2	34,368,861	Instituto Nacional de Estatística - Statistics Portugal: Agricultural Census 2009. Online data: http://ra09.ine.pt/xportal/xmain?xpid=RA2009&xpgid=ra_home
Serbia	2006	1	16,595,204	Statistical Office of Serbia: Municipalities of Serbia 2007 (GLIMS data warehouse)
Slovakia	2009	2	13,104,090	Statistical Office of the Slovak Republic: Livestock by territory, products and period. Online data: http://px-web.statistics.sk/PXWebSlovak/DATABASE/En/databasetree.asp
Slovenia	2010	1	4,005,852	Statistical Office of the Republic of Slovenia: Agricultural Census Slovenia 2010. Online data: http://www.stat.si/eng/novica_prikazi.aspx?id=4594
Spain	2009	2	178,328,136	Instituto Nacional de Estadística, Spain: Agrarian Census 2009. Online data: http://www.ine.es/jaxi/menu.do?type=pcaxis&path=%2Ft01%2Fp042/E01&file=inebase&L=1
Sweden	2009	1	7,158,272	Statistics Sweden: Yearbook of Agricultural Statistics 2010 (GLIMS data warehouse)
Switzerland	2009	1	8,741,117	Swiss Federal Statistical Office: Recensement des entreprises agricoles,1999-2009 (GLIMS data warehouse)
The former Yugoslav Republic of Macedonia	2008	1	2,226,055	State Statistic Office of the Republic of Macedonia: Total number of livestock, poultry and bee-hives (2007 and 2008) (GLIMS data warehouse)
United Kingdom	2009	2	131,354,251	Office for National Statistics, U.K.: Statistics by Region. Online data: http://www.statistics.gov.uk/hub/regional-statistics/

No Data for: Andorra, Bosnia and Herzegovina, Faroe Islands, Gibraltar, Guernsey, Holy See, Isle of Man, Jersey, Madeira Islands, Monaco, San Marino, Svalbard and Jan Mayen Islands

North America Tile

COUNTRY	YEAR	LEVEL	Totals in GLIMS	SOURCE
Belize	2002	1	1,764,435	Ministry Of Agriculture, Fisheries and Cooperatives, Belize: Annual Report 2002. Online data: http://www.agriculture.gov.bz/PDF/2002_annual_report.pdf
Canada	2006	2	118,641,120	Statistics Canada: 2006 Census of Agriculture. Online data: http://www.statcan.gc.ca/ca-ra2006/index-eng.htm
El Salvador	2007	2	35,123,656	Ministerio de Economía - El Salvador: IV Censo Agropecuario 2007-2008 (GLIMS data warehouse)
Guatemala	2003	2	21,518,212	Instituto Nacional de Estadística, Guatemala: IV Censo Nacional Agropecuario 2003. Online data: http://www.ine.gob.gt/np/agropecuario/tomo%20IV.pdf
Jamaica	2009	1	11,026,836	Agricultural Business Information System, Jamaica: Livestock Summary Report, 2009 (GLIMS data warehouse)
Martinique	2000	1	407,811	Agreste la Statistique Agricole, Le Ministère de l'Agriculture, de l'Alimentation, de la Pêche et de la Ruralité, France: Recensement Agricole 2000. Online data: http://agreste.agriculture.gouv.fr/recensement-agricole-2010/resultats-donnees-chiffrees/
Mexico	2007	2	154,126,180	Instituto Nacional de Estadística Geografía e Informática, México: Censo Agrícola, Ganadero y Forestal 2007. Online data: http://www.inegi.org.mx/est/contenidos/proyectos/Agro/ca2007/Resultados_Agricola/default.aspx
Nicaragua	2001	2	8,422,682	Instituto Nacional de Estadística y Censos, Nicaragua: III Censo Nacional Agropecuario 2001 (GLIMS data warehouse)
Panama	2001	2	14,082,185	Instituto Nacional de Estadística y Censo: Existencia de Animales en la República, por clase de animal, según provincia: 21 de abril de 1991 al 22 abril de 2001. Online data: http://www.contraloria.gob.pa/inec/Avance/Avance.aspx?ID_CATEGORIA=2&ID_CIFRAS=8&ID_IDIOMA=1
Puerto Rico	2007	2	5,031,810	National Agricultural Statistics Service - United States Department of Agriculture: National Agricultural Census 2007. Online data: http://www.agcensus.usda.gov/Publications/2007/Full_Report/
Saint Lucia	2007	6	129,861	Ministry of Agriculture, Forestry and Fisheries: Census of Agriculture - Final report 2007. Online data: http://www.govt.lc/www/publications/AgricultureCensus2007.pdf
Saint Vincent and the Grenadines	2000	1	65,920	Ministry of Agriculture and Labour, Government of Saint Vincent and the Grenadines: National Agricultural Census 2000 (GLIMS data warehouse)
United States of America	2007	2	1,692,431,565	National Agricultural Statistics Service - United States Department of Agriculture: National Agricultural Census 2007. Online data: http://www.agcensus.usda.gov/Publications/2007/Full_Report/

No Data for: Anguilla, Antigua and Barbuda, Bahamas, Barbados, Bermuda, Bird Island, British Virgin Islands, Cayman Islands, Costa Rica, Cuba, Dominica, Dominican Republic, Grenada, Guadeloupe, Haiti, Honduras, Montserrat, Netherlands Antilles, Saint Kitts and Nevis, Turks and Caicos Islands, United States Virgin Islands

South America Tile

COUNTRY	YEAR	LEVEL	Totals in GLIMS	SOURCE
Argentina	2002	2	244,131,110	Instituto Nacional de Estadística y Censos. Argentina: Censo Nacional Agropecuario 2002 (CNA 2002). Online data: http://www.indec.gov.ar/agropecuario/cna_principal.asp
Bolivia, Plurinational State of	2009	1	161,268,733	Instituto Nacional de Estadística, Bolivia: Existencia Total de Aves Parrilleras por Ano, Según Departamento 2000-2009 (GLIMS data warehouse)
Brazil	2009	2	1,024,999,717	Istituto Brasileiro de Geografia e Estatistica: Producao da Pecuria Municipal 2009. Online data: http://www.ibge.gov.br/home/estatistica/economia/ppm/2009/default.shtm
Chile	2007	1	40,362,000	Instituto Nacional de Estadísticas, Chile: Censo Agropecuario y Forestal 2007. Online data: http://www.ine.cl/canales/chile_estadistico/censos_agropecuarios/censo_agropecuario_07_comunas.php
Colombia	2009	1	144,913,543	Instituto Agropecuario, Colombia: Censo Agropecuario Nacional 2004 - 2009 (GLIMS data warehouse)
Ecuador	2000	2	40,823,593	Project SICA, Agricultural Information System, Ministry of Agriculture and Livestock, Ecuador: III Censo Nacional Agropecuario (GLIMS data warehouse)
French Guiana	1994	6	398,988	Institut National de la Statistique et des Etudes Economiques (INSEE): Tableaux Economiques Régionaux. Guyane 1995. Online data: http://www.insee.fr/fr/regions/guyane/collection.asp?id=76
Paraguay	2008	2	16,055,854	Ministerio de Agricultura y Ganadería, Paraguay: Censo Agropecuario Nacional 2008 (GLIMS data warehouse)
Peru	2007	1	120,226,000	Instituto Nacional de Estadística e Informática, Peru: Compendio Estadístico 2007 (GLIMS data warehouse)
Suriname	1994	1	6,459,453	Environmental Research Group Oxford, ERGO: ERGO, 1996 "Livestock Geography. A demonstration of GIS techniques applied to Global Livestock Systems and Populations", Animal Health Division, FAO, Rome (William Wint extracted from the agroecological zone estimates derived from the FAOSTAT data for 1994) (GLIMS data warehouse)
Uruguay	2000	1	9,912,543	Instituto Nacional de Estadística, Republica Oriental del Uruguay: Censo Agropecuario 2000 (GLIMS data warehouse)
Venezuela, Bolivarian Republic	2007	1	91,417,167	Ministerio del Poder Popular para la Agricultura y Tierras - Venezuela: VII Censo Agrícola 2007. Online data: http://censo.mat.gob.ve/

No Data for: Aruba, Falkland Islands, Guyana, Trinidad and Tobago

Oceania Tile

COUNTRY	YEAR	LEVEL	Totals in GLIMS	SOURCE
American Samoa	2003	1	35,709	National Agricultural Statistics Service - United States Department of Agriculture: National Agricultural Census 2007. Online data: http://www.agcensus.usda.gov/Publications/2007/Full_Report/
Australia	2007	6	94,199,025	Australian Bureau of Statistics: Agricultural commodities: small area data, Australia 2006-2007 (GLIMS data warehouse)
Cook Islands	2006	1	30,611	Statistic Office: Census of Population Dwellings (2001 and 2006) (GLIMS data warehouse)
New Caledonia	2002	1	344,176	Institut de la Statistique et des Etudes Economiques (ISEE): Recensement Général Agricole (RGA) 2002. Online data: http://www.davar.gouv.nc/portal/page/portal/davar/secteur_rural/statistiques_agricoles/donnees_synthese/recensement_agricole
Northern Mariana Islands	2007	1	11,812	National Agricultural Statistics Service - United States Department of Agriculture: National Agricultural Census 2007. Online data: http://www.agcensus.usda.gov/Publications/2007/Full_Report/
Tonga	2001	1	177,829	Ministry of Agriculture and Forestry, Kingdom of Tonga: Agriculture Census 2001. Online data: http://www.spc.int/prism/tonga/index.php/surveys/agriculture-census-2011

No Data for: Ashmore and Cartier Islands, Fiji, French Polynesia, Guam, Kiribati, Marshall Islands, Federated States of Micronesia, Nauru, New Zealand, Niue, Norfolk Island, Palau, Papua New Guinea, Pitcairn, Samoa, Solomon Islands, Tokelau, Tuvalu, Vanuatu, Wake Island, Wallis and Futuna

Supplementary information 5d – Country details on sub-national statistics for ducks (year and level of observation) by continental tile.

Africa Tile (Data insufficient to run the regional model)

COUNTRY	YEAR	LEVEL	Totals in GLIMS	SOURCE
Burkina Faso	2003	2	211,828	Ministère des Ressources Animales: Les Statistiques du Secteur de l'Élevage au Burkina Faso, 2003 & 2008 (GLIMS data warehouse)
Cameroon	1999	1	194,579	Direction des Etudes, de la Statistique et de la Coopération/MINEPIA (1999) et Institut National de la Statistique (1998): Revue du sector avicole (GLIMS data warehouse)
Congo	2000	1	1,023	Ministère de l'Agriculture et de l'Élevage: Enquête Agricole Pilote, Campagne Agricole 1999-2000 (GLIMS data warehouse)
Egypt	2011	1	4,658,920	Central Agency for Public Mobilization and Statistics, Egypt: Estimated numbers of livestock and animals by Governorate 2011 (GLIMS data warehouse)
Ghana	2009	1	330,045	CountrySTAT - Food and agriculture data network (GLIMS data warehouse)
Guinea	2001	1	387,482	Service National des Statistiques Agricoles - Ministère de l'Agriculture, de l'Élevage et des Forêts: Recensement national de l'agriculture, Campagne agricole 2000-2001 (GLIMS data warehouse)
Guinea-Bissau	1994	2	42,988	Instituto Nacional de Estadística: Inquérito Anual sobre Superficie Rendimento e Producao. Campanha 93/94 (GLIMS data warehouse)
Madagascar	2005	2	24,213,521	Ministère de l'Agriculture, Secrétariat General, Direction de la Prévision et des Etudes Economiques, Madagascar: Recensement de l'agriculture (RA) - Campagne agricole 2004-2005 (GLIMS data warehouse)
Malawi	2007	2	429,170	Agriculture Statistics Division of the National Statistical Office of Malawi: National Census of Agriculture and Livestock (October 2006 - October 2007) (GLIMS data warehouse)
Mali	2004	1	161,269	Ministère de l'Agriculture de l'Élevage et de la Pèche, République du Mali: Recensement de l'Agriculture 2004-2005 (GLIMS data warehouse)
Mozambique	2010	1	1,881,736	National Directorate of Livestock: Censo AgroPecuario 2009-2010 (GLIMS data warehouse)
Niger	2007	1	427,747	Ministère des Ressources Animales: Recensement Général de l'Agriculture et du Cheptel (RGAC 2005/2007). Online data: http://harvestchoice.org/publications/niger-recensement-general-de-lagriculture-et-du-cheptel-rgac-20052007-volume-v-producti
Nigeria	2011	1	9,553,912	National Bureau of Statistics, Federal Ministry of Agriculture and Rural Development: National Agriculture Sample Survey 2010/2011 (GLIMS data warehouse)
Rwanda	2008	1	6,729	Rwanda Animal Resources Development Authority (RARDA) (GLIMS data warehouse)
Sao Tome and Principe	1997	2	8,713	Ministère de l'Agriculture et de la Pêche: Enquête Agricole de Production (EAP) 1997 (GLIMS data warehouse)

Sierra Leone	2004	2	487,508	Ministry of Agriculture & Food Security of Sierra Leone: 2004 Populations and Housing Census Statistical Office (GLIMS data warehouse)
Togo	2005	1	13,200	Division de la Production et de la Santé Animales de la FAO, Centre d'Urgence pour les Maladies Animales Transfrontaliers, Unité de Socio-Économie, Production et Biodiversité. Revue du secteur avicole: Togo, 2008 (GLIMS data warehouse)
Uganda	2000	4	671,813	Uganda Bureau of Statistics: Uganda Census 2000 (GLIMS data warehouse)
United Republic of Tanzania	2008	2	1,156,726	Ministry of Water and Livestock Development of Tanzania: National Sample Census of Agriculture, 2007/2008 (GLIMS data warehouse)

No Data for: Algeria, Angola, Bassas da India, Benin, Botswana, British Indian Ocean Territory, Burundi, Côte d'Ivoire, Cape Verde, Central African Republic, Chad, Comoros, Democratic Republic of the Congo, Djibouti, Equatorial Guinea, Eritrea, Ethiopia, Europa Island, Gabon, Gambia, Glorioso Island, Juan de Nova Island, Kenya, Lesotho, Liberia, Libya, Mauritania, Mauritius, Mayotte, Morocco, Namibia, Réunion, Saint Helena, Senegal, Seychelles, Somalia, South Africa, Sudan, Swaziland, Tromelin Island, Tunisia, Western Sahara, Zambia, Zimbabwe

Asia Tile

COUNTRY	YEAR	LEVEL	Totals in GLIMS	SOURCE
Afghanistan	2003	2	387,038	Ministry of Agriculture, Irrigation and Livestock of Afghanistan: Afghanistan - National Livestock Census 2002-2003 - Final Report. Online data: http://reliefweb.int/sites/reliefweb.int/files/resources/58C374339FC582F149256DF30009D60F-fao-afg-4dec.pdf
Bangladesh	2005	6	37,932,995	Bangladesh Bureau of Statistics: Data Retrieved 12 November 2009 from http://www.bbs.gov.bd/ (GLIMS data warehouse)
Belarus	2005	1	1,017,938	Ministry of Statistics and Analysis of The Republic of Belarus: Statistical Yearbook of the Republic of Belarus 2005 (GLIMS data warehouse)
Cambodia	2009	2	5,122,207	Department of Animal Production and Health, Cambodia: 2009 district data (David Bourn, "Environmental Animal Health Management Initiative for Enhanced Smallholder Production", 2010) (GLIMS data warehouse)
China	2005	6	931,845,945	Editorial Department of China Animal Husbandry: China animal husbandry yearbook. Beijing, China: China Agricultural Press (GLIMS data warehouse)
India	2007	2	27,154,998	Department of Animal Husbandry, Dairying & Fisheries, Ministry of Agriculture, India: Census of Agriculture, 2007 (GLIMS data warehouse)
Indonesia	2010	1	44,301,804	Department Pertanian Republik Indonesia: Buku Statistik, 2011 (GLIMS data warehouse)
Lao People's Democratic Republic	2009	2	6,279,320	Department of Livestock and Fisheries, Laos: 2009 district data (David Bourn, " Environmental Animal Health Management Initiative for Enhanced Smallholder Production" , 2010) (GLIMS data warehouse)
Malaysia	2001	1	6,013,621	Department of Veterinary Service, Malaysia: Livestock Products statistics 1996-2002 (GLIMS data warehouse)
Myanmar	2006	6	9,745,621	Livestock Breeding and Veterinary Department (LBVD), Ministry of Livestock Breeding and Fisheries, Yangon, Myanmar: Myanmar Animal Census 2006 (GLIMS data warehouse)
Nepal	2000	3	425,158	Ministry of Agriculture and Co-operatives, Agri-Business and Statistics Division (GLIMS data warehouse)
Pakistan	1996	2	1,127,108	Agricultural Census Organization, Ministry of Food & Agriculture, Pakistan: Livestock census 1996 - special report. Lahore, Pakistan (GLIMS data warehouse)
Philippines	2006	3	7,739,879	Bureau of Animal Industry, Philippines: 2006 data (David Bourn, " Environmental Animal Health Management Initiative for Enhanced Smallholder Production" , 2010) (GLIMS data warehouse)
Qatar	2001	1	12,266	Ministry of Municipal Affairs and Agriculture Department of Agriculture and Water Research: Agricultural Census 2000-2001 (GLIMS data warehouse)
Republic of Korea	2004	1	8,265,580	Korea National Statistical Office: Korea Statistical Yearbook 2005 (GLIMS data warehouse)

Romania	2002	6	3,897,538	EUROSTAT on line database. Section: General Statistics, Region, Agriculture, Animal Populations and Land Use. February 2005. Online data: http://epp.eurostat.ec.europa.eu/portal/page/portal/agriculture/data/
Russian Federation	2006	1	148,273	Russian Federation - Federal State Statistical Service: Results of All-Russia Agricultural Census 2006 (1-8 vol.) (GLIMS data warehouse)
Sri Lanka	2002	2	23,940	Agriculture and Environment Statistics Division, Department of Census and Statistics, Sri Lanka: Census of Agriculture 2002, All Sectors. Online data: http://www.statistics.gov.lk/agriculture/AGC2002/AGC2002.htm
Thailand	2004	3	32,620,489	Department of Livestock Development, Ministry of Agriculture and Cooperatives, Thailand: Census 2004 (X- ray surveys searching for HPAI outbreaks) (GLIMS data warehouse)
Turkey	2009	2	413,523	Turkish Statistical Institute: Livestock statistics database 2009 (GLIMS data warehouse)
Ukraine	2009	1	8,743,007	State Statistics Committee Ukraine: Head of livestock and poultry by regions (GLIMS data warehouse)
Viet Nam	2001	6	60,758,232	General Statistics Office of Vietnam: Agricultural Census database 2001. Online data: http://www.gso.gov.vn/default_en.aspx?tabid=477&idmid=4&ItemID=1824

No Data for: Armenia, Azerbaijan, Bahrain, Bhutan, Brunei Darussalam, Bulgaria, Christmas Island, Cocos (Keeling) Islands, Cyprus, Democratic People's Republic of Korea, Georgia, Islamic Republic of Iran, Iraq, Israel, Japan, Jordan, Kazakhstan, Kuril Islands, Kuwait, Kyrgyzstan, Lebanon, Liancourt Rock, Maldives, Mongolia, Oman, Paracel Islands, Republic of Moldova, Saudi Arabia, Scarborough Reef, Senkaku Islands, Singapore, Spratly Islands, Syrian Arab Republic, Tajikistan, Timor-Leste, Turkmenistan, United Arab Emirates, Uzbekistan, Yemen

Europe Tile

COUNTRY	YEAR	LEVEL	Totals in GLIMS	SOURCE
Albania	2010	1	164,500	Institute of Statics, Albania: Agricultural sector, Livestock 2001-2010 (GLIMS data warehouse)
Austria	2010	1	3,134,156	Statistics Austria: Livestock Survey, 2010 (GLIMS data warehouse)
Belgium	2010	2	6,429,600	EUROSTAT online database. General Statistics, Region, Agriculture, Animal Populations and Land Use. Online data: http://epp.eurostat.ec.europa.eu/portal/page/portal/agriculture/data/
Bosnia and Herzegovina	2009	6	529,095	Federal Office of Statistics of Federation of Bosnia and Herzegovina: Number of animals from Federal Agro-Mediterranean Institute of Mostar (GLIMS data warehouse)
Croatia	2003	1	1,726,895	Central Bureau of Statistics, Republic of Croatia: Agricultural Census 2003. Online data: http://www.dzs.hr/default_e.htm
Czech Republic	2010	2	1,906,929	Czech Statistical Office: Agrocensus regions: Farm Structure Survey and Survey on Agricultural Production Methods 2010. Online data: http://www.czso.cz/csu/2012edicniplan.nsf/engp/2127-12
Denmark	2010	2	13,173,060	Denmark Statistics: Farms by region and selected farms, crops and livestock. Online data: http://www.statistikbanken.dk/statbank5a/SelectVarVal/Define.asp?Maintable=BDF51&PLanguage=1
Estonia	2001	2	327,195	Statistikaamet Statistical Office of Estonia: Agricultural Census 2001. Online data: http://www.stat.ee/agricultural-census-2001
Finland	2010	6	1,366,800	The Information Centre of the Ministry of Agriculture and Forestry, Finland: Yearbook of Farm Statistics 2011 (GLIMS data warehouse)
France	2009	2	13,245,288	Service de la Statistique et de la Prospective (SSP): Statistique Agricole Annuelle (GLIMS data warehouse)
Germany	2007	2	25,247,881	Federal Statistical Office, Germany: GENESIS database. Online data: https://www.regionalstatistik.de/genesis/online/online;jsessionid=EE45147898822814978BE734145275C4?operation=sprachwechsel&option=en
Greece	2007	2	1,111,540	Hellenic Statistic Authority (EL.STAT): Farms and number of animals by type, region and county (GLIMS data warehouse)
Hungary	2010	1	3,169,000	Hungarian Central Statistical Office: Regional Statistics. Online data: http://www.ksh.hu/agriculture
Iceland	2010	2	3,615	Statistics Iceland: 2010 data (GLIMS data warehouse)
Ireland	2000	1	1,722,108	Central Statistical Office Ireland: Census of Agriculture 2000. Online data: http://www.cso.ie/en/releasesandpublications/agricultureandfishing/censusofagriculturemainresultsjune2000/
Italy	2010	3	9,331,143	Istituto Nazionale di Statistica: Censimento Agricoltura 2010. Online data: http://dati-censimentoagricoltura.istat.it
Latvia	2001	1	368,909	Central Statistical Bureau of Latvia: Results of 2001 Agricultural Census. Online data: http://www.csb.gov.lv/en/dati/agricultural-census-2001-30765.html

Liechtenstein	2009	6	1,811	Amt für Statistik Liechtenstein: Landwirtschaftsstatistik 2009 (GLIMS data warehouse)
Lithuania	2010	2	942,163	Lietuvos Statistikas Departamenta: Number of livestock by administrative territory, 2011 (GLIMS data warehouse)
Luxembourg	2008	2	77,841	Portail des statistiques du Grand-Duché de Luxembourg (STATEC): Recensement Agricole 2008 (GLIMS data warehouse)
Malta	2010	6	70,593	National Statistics Office of Malta: Census of Agriculture 2010. Online data: http://www.nso.gov.mt/statdoc/document_view.aspx?id=3287&backurl=/themes/theme_page.aspx
Montenegro	2010	1	41,118	Statistical office of Montenegro (MONSTAT): Agriculture Census 2010. Online data: http://www.monstat.org/eng/page.php?id=58&pageid=58
Netherlands	2010	2	12,254,972	Centraal Bureau voor de Statistiek, Netherlands: Agriculture: crops, livestock and land use by general far type, region - 2011 (GLIMS data warehouse)
Norway	2010	1	1,607,772	Statistics Norway: Agriculture Statistics 2011 (GLIMS data warehouse)
Poland	2009	1	14,278,600	Central Statistical Office, Poland: Statistical Yearbook of Agriculture 2010 (GLIMS data warehouse)
Portugal	2009	2	1,851,290	Instituto Nacional de Estatística - Statistics Portugal: Agricultural Census 2009. Online data: http://ra09.ine.pt/xportal/xmain?xpid=RA2009&xpgid=ra_home
Serbia	2006	1	3,998,927	Statistical Office of Serbia: Municipalities of Serbia 2007 (GLIMS data warehouse)
Slovakia	2009	2	740,862	Statistical Office of the Slovak Republic: Livestock by territory, products and period. Online data: http://px-web.statistics.sk/PXWebSlovak/DATABASE/En/databasetree.asp
Slovenia	2010	2	308,540	Statistical Office of the Republic of Slovenia: Agricultural Census Slovenia 2010. Online data: http://www.stat.si/eng/novica_prikazi.aspx?id=4594
Spain	2010	2	25,704,041	Ministerio de Medio Ambiente y Medio Rural y Marino, Gobierno de España (MARM): Encuestas Ganaderas, 2010 (GLIMS data warehouse)
Sweden	2009	1	1,528,740	Statistics Sweden: Yearbook of Agricultural Statistics 2010 (GLIMS data warehouse)
Switzerland	2009	1	1,557,204	Swiss Federal Statistical Office: Recensement des entreprises agricoles,1999-2009 (GLIMS data warehouse)
The former Yugoslav Republic of Macedonia	2006	2	173,185	State Statistic Office of the Republic of Macedonia: Census of Agriculture 2007. Online data: http://www.stat.gov.mk/PrikaziPublikacija_en.aspx?id=51&rbr=205
United Kingdom	2009	2	4,525,710	Office for National Statistics, U.K.: Statistics by Region. Online data: http://www.statistics.gov.uk/hub/regional-statistics/

No Data for: Andorra, Faroe Islands, Gibraltar, Guernsey, Holy See, Isle of Man, Jersey, Madeira Islands, Monaco, San Marino, Svalbard and Jan Mayen Islands

North America Tile

COUNTRY	YEAR	LEVEL	Totals in GLIMS	SOURCE
Belize	2003	1	21,224	Ministry Of Agriculture, Fisheries and Cooperatives, Belize: Annual Report 2003. Online data: http://www.agriculture.gov.bz/PDF/Annual_Report_2003.pdf
Canada	2006	2	14,168,313	Statistics Canada: 2006 Census of Agriculture. Online data: http://www.statcan.gc.ca/ca-ra2006/index-eng.htm
Cayman Islands	2009	1	1,063	Economics and Statistics Office - Government of the Cayman Islands: Statistical Compendium 2009 (GLIMS data warehouse)
Costa Rica	2006	1	297,359	Ministerio de Agricultura y Ganadería, Costa Rica: Programa Nacional de Sanidad Porcina 2006 (GLIMS data warehouse)
El Salvador	2007	2	165,866	Ministerio de Economía - El Salvador: IV Censo Agropecuario 2007-2008 (GLIMS data warehouse)
Guadeloupe	2000	1	25,839	Agreste la Statistique Agricole, Le Ministère de l'Agriculture, de l'Alimentation, de la Pêche et de la Ruralité, France: Recensement Agricole 2000. Online data: http://agreste.agriculture.gouv.fr/recensement-agricole-2010/resultats-donnees-chiffrees/
Guatemala	2003	2	419,170	Instituto Nacional de Estadística, Guatemala: IV Censo Nacional Agropecuario 2003. Online data: http://www.ine.gob.gt/np/agropecuario/tomo%20IV.pdf
Jamaica	2009	1	361,540	Agricultural Business Information System, Jamaica: Livestock Summary Report, 2009 (GLIMS data warehouse)
Martinique	2000	1	20,621	Agreste la Statistique Agricole, Le Ministère de l'Agriculture, de l'Alimentation, de la Pêche et de la Ruralité, France: Recensement Agricole 2000. Online data: http://agreste.agriculture.gouv.fr/recensement-agricole-2010/resultats-donnees-chiffrees/
Mexico	2007	2	8,964,101	Instituto Nacional de Estadística Geografía e Informática, México: Censo Agrícola, Ganadero y Forestal 2007. Online data: http://www.inegi.org.mx/est/contenidos/proyectos/Agro/ca2007/Resultados_Agricola/default.aspx
Nicaragua	2001	2	383,172	Instituto Nacional de Estadística y Censos, Nicaragua: III Censo Nacional Agropecuario, 2001 (GLIMS data warehouse)
Panama	2001	2	312,102	Instituto Nacional de Estadística y Censo: Existencia de animales en la República, por clase de animal, según provincia: 21 de abril de 1991 al 22 abril de 2001. Online data: http://www.contraloria.gob.pa/inec/Avance/Avance.aspx?ID_CATEGORIA=2&ID_CIFRAS=8&ID_IDIOMA=1
Puerto Rico	2007	2	63,585	National Agricultural Statistics Service - United States Department of Agriculture: National Agricultural Census 2007. Online data: http://www.agcensus.usda.gov/Publications/2007/Full_Report/

Saint Lucia	2007	6	19,520	Ministry of Agriculture, Forestry and Fisheries: Census of Agriculture - Final report 2007. Online data: http://www.govt.lc/www/publications/AgricultureCensus2007.pdf
Saint Vincent and the Grenadines	2000	1	4,807	Ministry of Agriculture and Labour, Government of Saint Vincent and the Grenadines: National Agricultural Census 2000 (GLIMS data warehouse)
United States of America	2007	2	67,239,043	National Agricultural Statistics Service - United States Department of Agriculture: National Agricultural Census 2007. Online data: http://www.agcensus.usda.gov/Publications/2007/Full_Report/

No Data for: Anguilla, Antigua and Barbuda, Bahamas, Barbados, Bermuda, Bird Island, British Virgin Islands, Cuba, Dominica, Dominican Republic, Grenada, Haiti, Honduras, Montserrat, Netherlands Antilles, Saint Kitts and Nevis, Turks and Caicos Islands, United States Virgin Islands

South America Tile (Data insufficient to run the regional model)

COUNTRY	YEAR	LEVEL	Totals in GLIMS	SOURCE
Colombia	2001	1	546,272	Ministerio de Agricultura y Desarrollo Rural, Departamento Administrativo Nacional de Estadística, Colombia: Encuesta Nacional Agropecuaria - Resultados 2001 (GLIMS data warehouse)
Ecuador	2000	2	351,945	Project SICA, Agricultural Information System, Ministry of Agriculture and Livestock, Ecuador: III Censo Nacional Agropecuario (GLIMS data warehouse)
Paraguay	2001	1	717,600	Dirección General de Estadística, Encuestas y Censos: Anuario Estadístico 2001 (GLIMS data warehouse)
Venezuela, Bolivarian Republic	2007	1	142,724	Ministerio del Poder Popular para la Agricultura y Tierras - Venezuela: VII Censo Agrícola 2007. Online data: http://censo.mat.gob.ve/

No Data for: Argentina, Aruba, Plurinational State of Bolivia, Brazil, Chile, Falkland Islands (Malvinas), French Guiana, Guyana, Peru, Suriname, Trinidad and Tobago, Uruguay

Oceania Tile

COUNTRY	YEAR	LEVEL	Totals in GLIMS	SOURCE
Australia	2007	6	3,042,342	Australian Bureau of Statistics: Agricultural commodities: small area data, Australia 2006-2007 (GLIMS data warehouse)
Cook Islands	2006	1	436	Statistic Office: Census of Population Dwellings (2001 and 2006) (GLIMS data warehouse)
Fiji	2009	2	72,486	Department of Agriculture Economic Planning and Statistic Division: National Agricultural Census 2009 report . Online data : http://www.fao.org/fileadmin/templates/ess/ess_test_folder/World_Census_Agriculture/Country_info_2010/Reports/Reports_3/FJI_ENG_REP_2009.pdf
New Caledonia	2002	2	9,844	Institut de la Statistique et des Etudes Economiques (ISEE): Recensement Général Agricole (RGA) 2002. Online data: http://www.davar.gouv.nc/portal/page/portal/davar/secteur_rural/statistiques_agricoles/donnees_synthese/recensement_agricole
Northern Mariana Islands	2007	1	189	National Agricultural Statistics Service - United States Department of Agriculture: National Agricultural Census 2007. Online data: http://www.agcensus.usda.gov/Publications/2007/Full_Report/
Tonga	2001	1	1,119	Ministry of Agriculture and Forestry, Kingdom of Tonga: Agriculture Census 2001. Online data: http://www.spc.int/prism/tonga/index.php/surveys/agriculture-census-2011
