# Supplementary material for: Mapping the Global Distribution of Livestock
Source: PLoS One. 2014 May 29;9(5):e96084. doi: 10.1371/journal.pone.0096084 (PMC4038494; doi:10.1371/journal.pone.0096084)
Supplement: Information S6 — 6a. Average Spatial Resolution (ASR) of the training data used to model a) cattle; b) pigs; c) chickens and d) ducks distributions. The ASR measures the effective resolution of input administrative units in kilometers. It is calculated as the square root of the land area divided by the number of administrative units [1], [2]. (file SI6.pdf) Supplementary information 6b. Country level of detail for the training data of a) cattle; b) pigs; c) chickens and d) ducks. (PDF) [file pone.0096084.s006.pdf]

Supplementary information 6a – Average Spatial Resolution (ASR) of the training data used to model a) cattle; b) pigs; c) chickens and d) ducks distributions. The ASR measures the effective resolution of input administrative units in kilometers. It is calculated as the square root of the land area divided by the number of administrative units [1,2].

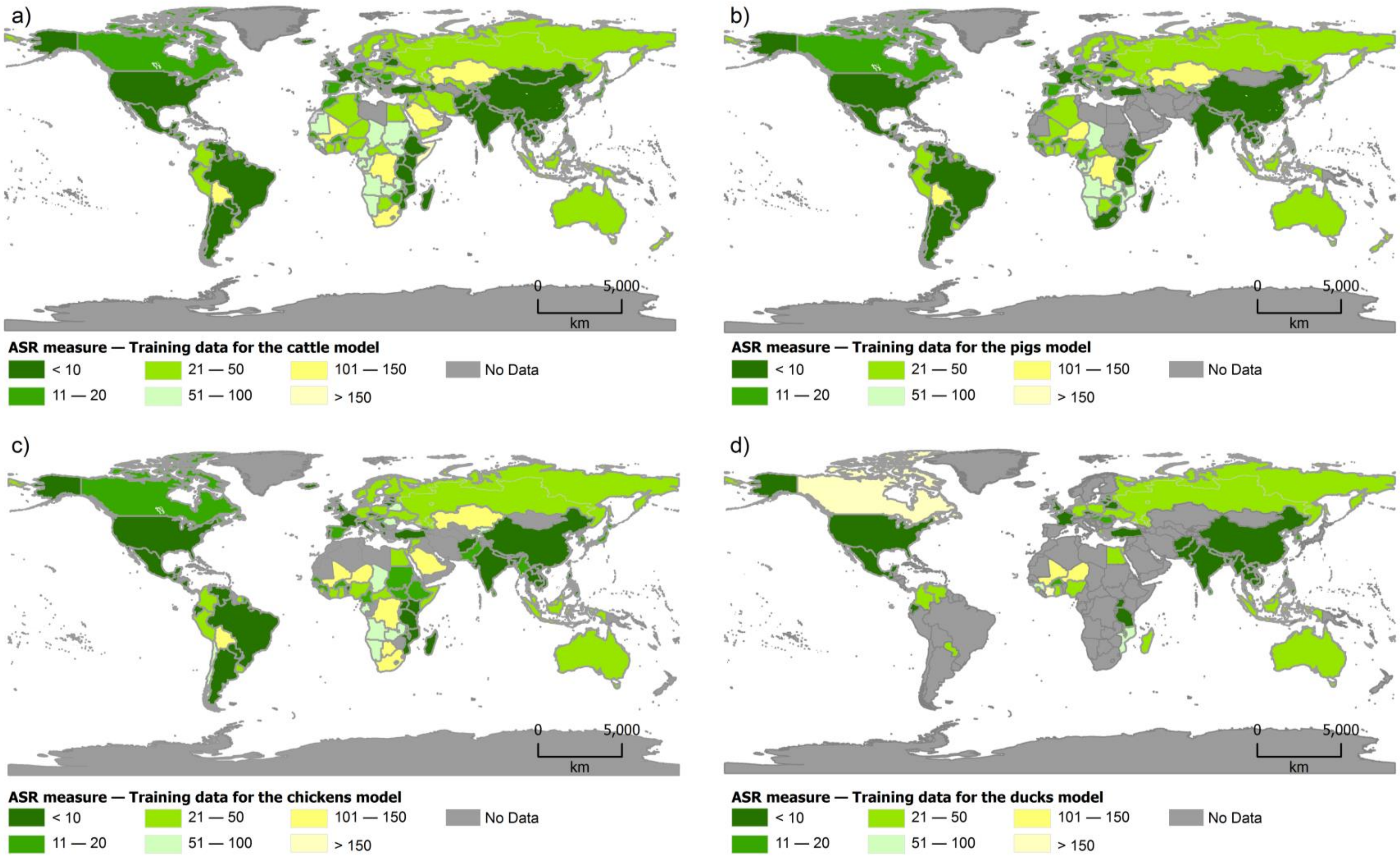

Supplementary information 6b – Country level of detail for the training data of a) cattle; b) pigs; c) chickens and d) ducks.

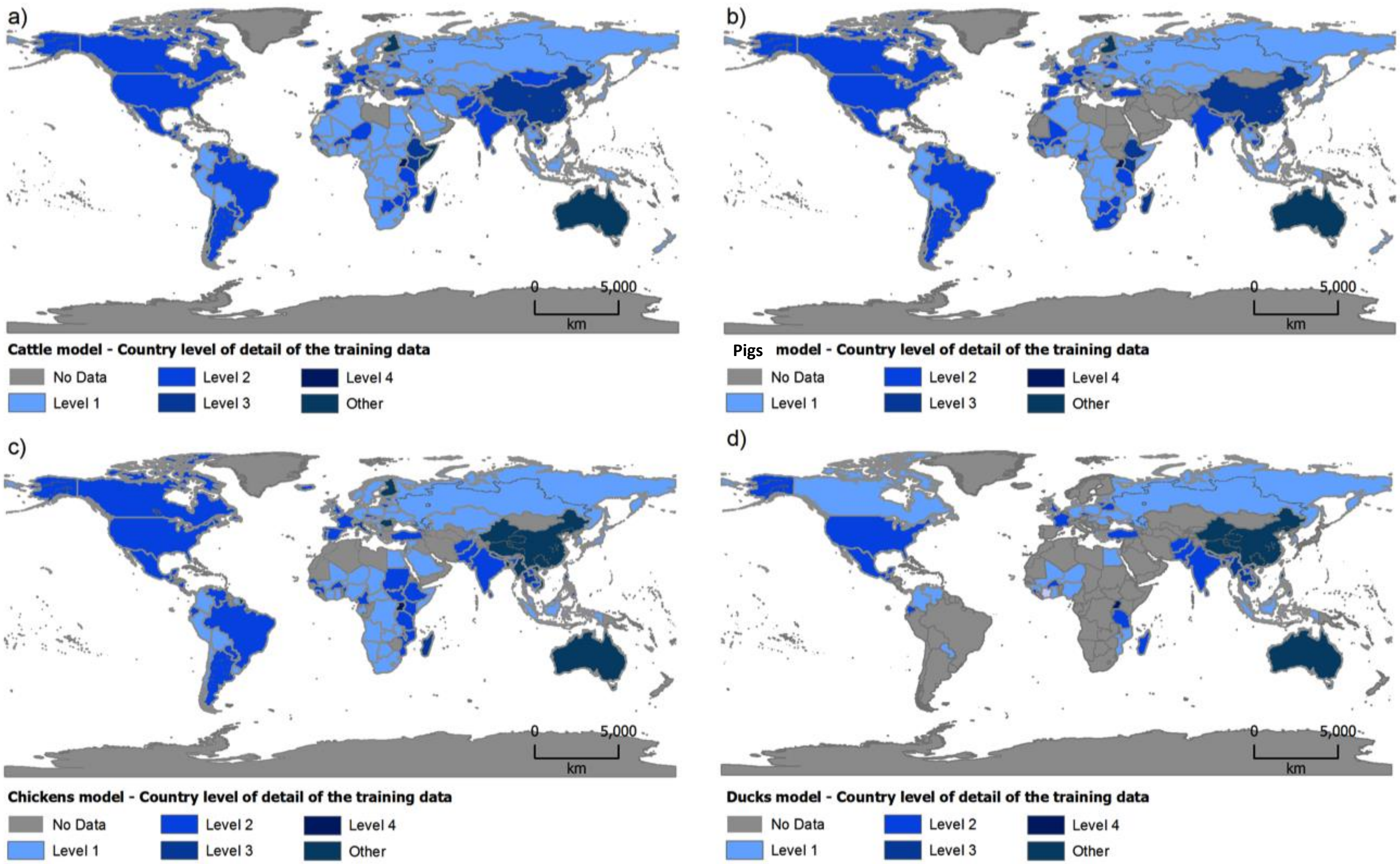

References

1. Balk D, G Y (2004) The Global Distribution of Population: Evaluating the gains in resolution refinement. Available at: http://sedac.ciesin.columbia.edu/downloads/docs/gpw-v3/gpw3_documentation_final.pdf. Accessed 2014 February 24.

2. Linard C, Gilbert M, Snow RW, Noor AM, Tatem AJ (2012) Population Distribution, Settlement Patterns and Accessibility across Africa in 2010. PLoS ONE 7: e31743.
