# Supplementary material for: Mapping the Global Distribution of Livestock
Source: PLoS One. 2014 May 29;9(5):e96084. doi: 10.1371/journal.pone.0096084 (PMC4038494; doi:10.1371/journal.pone.0096084)
Supplement: Information S7 — Polygon details and processing time for each continental tile and species. (PDF) [file pone.0096084.s007.pdf]

Supplementary information 7 – Polygon details and processing time for each continental tile and species.

Tile	Species	Total polygons	Polygons with no data	Polygons with 0 values*	Processing time in hours**
**Africa**	Cattle	7,658	836	922	98
	Chickens	6,760	300	28	57
	Pigs	7,992	308	2,253	30
	Ducks	n.a.	n.a.	n.a.	n.a.
**Asia**	Cattle	19,506	258	1,342	210
	Chickens	23,870	253	746	136
	Pigs	17,802	1,124	838	184
	Ducks	23,887	300	1,223	173
**Europe**	Cattle	13,857	91	1,391	48
	Chickens	9,741	34	3,499	25
	Pigs	9,981	149	3,290	25
	Ducks	636	128	7	17
**North America**	Cattle	6,902	173	37	114
	Chickens	6,827	475	258	144
	Pigs	6,839	658	270	139
	Ducks	6,308	867	2,111	54
**Oceania**	Cattle	1,744	51	26	27
	Chickens	1,747	128	--	46
	Pigs	275	51	9	18
	Ducks	1,760	50	154	25
**South America**	Cattle	7,217	145	81	71
	Chickens	6,914	591	--	128
	Pigs	7,217	133	148	64
	Ducks	n.a.	n.a.	n.a.	n.a.

* In the chicken model, polygons with 0 value are treated as no data in the training data file, on the assumption that at least some chickens are present in every polygon; zero values are more likely to be data gaps than reliable reports. **Models were run on 4 dedicated DELL machines with installed 64-bit Microsoft Windows Vista™ OS – 8 GB of RAM – and Intel(R) Core(TM)2 QUAD CPU.
