# Supplementary material for: Mapping the Global Distribution of Livestock
Source: PLoS One. 2014 May 29;9(5):e96084. doi: 10.1371/journal.pone.0096084 (PMC4038494; doi:10.1371/journal.pone.0096084)

Supplementary information 8 – Procedure to download the output raster data.

- Go to the Livestock Geo-Wiki on: http://www.livestock.geo-wiki.org;
- Click on "**Register here!**" in the right hand panel (or go directly to: http://www.geo-wiki.org/registration/);
- Enter your details, follow the instructions and log in;
- Click on "**Go to Application**" in the right hand panel;
- Verify that the top left drop-down menu is set on "**Livestock**" (see below);
- Different livestock distributions can be selected for viewing by clicking on the "**Livestock distributions**" button on the left hand menu (see below);
- Click on the "**Download**" button on the left hand menu;
- Data for each species are downloaded by clicking on their respective buttons and following the instructions;
- The metadata of each layer can be accessed by clicking on the "**metadata**" link.

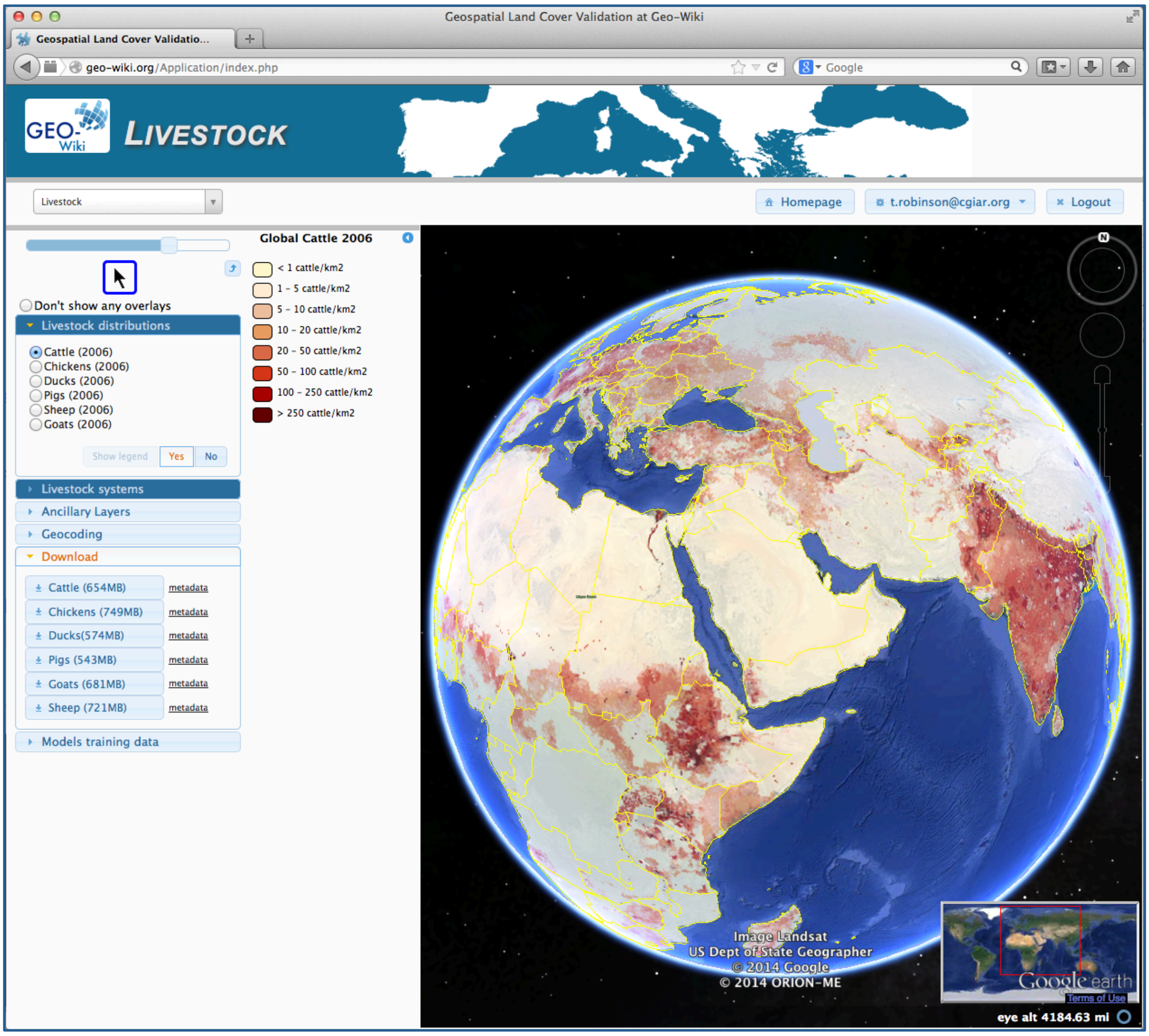

Supplement: Information S8 — Procedure to download the output raster data. (PDF) [file pone.0096084.s008.pdf]
